# Intracerebral Hemorrhage: The Global Differential Burden and Secular Trends From 1990 to 2019 and Its Prediction up to 2030

**DOI:** 10.3389/ijph.2025.1607013

**Published:** 2025-05-21

**Authors:** Xuesong Yang, Yanbo Liu, Shiling Chen, Danyang Chen, Xuan Wu, Yanqiong Wu, Cheng Liu, Anne Manyande, Hongbing Xiang, Zhouping Tang

**Affiliations:** ^1^ Department of Anesthesiology, Tongji Hospital, Tongji Medical College, Huazhong University of Science and Technology, Wuhan, Hubei, China; ^2^ Department of Neurology, Tongji Hospital, Tongji Medical College, Huazhong University of Science and Technology, Wuhan, Hebei, China; ^3^ School of Human and Social Sciences, University of West London, Middlesex, United Kingdom

**Keywords:** risk factor, intracerebral hemorrhage, global incidence, global mortality, global disability

## Abstract

**Objectives:**

This study aims to analyze the global burden and temporal trends of intracerebral hemorrhage from 1990 to 2019 and to project the burden up to 2030, considering variations across regions, sexes, and age groups.

**Methods:**

Data were sourced from the GBD (Global Burden of Disease) 2019 study. We assessed ASIR (age-standardized incidence rates), ASMR (age-standardized mortality rates) , and ASDR (age-standardized disability adjusted life year rate) using the BAPC (Bayesian age-period-cohort) model. Spearman's Rho correlation was used to examine the relationship between disease burden and the SDI (Socio-Demographic Index).

**Results:**

From 1990 to 2019, the global ASIR, ASMR, and ASDR of intracerebral hemorrhage decreased by 1.52%, 1.64%, and 1.64%, respectively, while absolute case numbers increased. Males consistently exhibited higher ASIR, ASMR, and ASDR than females. The projections suggest that by 2030, the incidence and absolute cases of intracerebral hemorrhage will continue to rise, while mortality rates will decline.

**Conclusion:**

Despite reductions in age-standardized rates, the global burden of intracerebral hemorrhage continues to increase due to population growth and aging. Effective prevention and treatment strategies, especially in low-SDI regions, are urgently needed.

## Introduction

Cerebrovascular diseases represent a collective term for a group of disorders resulting from cerebrovascular lesions that can lead to cerebral functional impairment [1]. Among them, stroke is the primary clinical subtype of cerebrovascular diseases, which include ischemic stroke and hemorrhagic stroke [2]. It is characterized by the sudden onset of focal or diffuse cerebral functional deficits and constitutes a cluster of organic brain injuries attributed to cerebrovascular pathology [3]. Hemorrhagic stroke, in particular, deserves heightened attention due to its elevated mortality, disability rate, and poor prognosis. Preliminary investigations have revealed an acute-phase fatality rate of approximately 30%–40% for intracerebral hemorrhage [4, 5]. The principal etiological factor contributing to intracerebral hemorrhage is the coexistence of high blood pressure and hypertensive small vessel disease.

Chronic hypertension induces arteriolar hyalinosis and fibrinoid necrosis in the cerebral microvasculature, rendering them susceptible to rupture when exposed to abrupt blood pressure elevations [6]. The majority of hypertensive-related hemorrhagic strokes occur in the basal ganglia, with other common bleeding sites such as cerebral lobes, brainstem, and cerebellum [7]. Hypertension-related ischemic strokes most frequently involve deep penetrating branches of the middle cerebral artery, the brainstem arteries, and thalamoperforating branches of the posterior cerebral artery [8]. Cerebral hemorrhage often exhibits increased incidence during cold seasons [9], with patients typically experiencing emotional agitation or physical activity at the time of onset [10]. Patients commonly present with markedly elevated blood pressure after onset, specific neurological deficits attributed to hematoma compression and increased intracranial pressure, as well as varying degrees of headache, vomiting, and altered consciousness [11]. Prior research has established associations between hemorrhagic stroke and risk factors such as hypertension, smoking, and alcohol consumption [12]. However no study so far, based on the GBD 2019 study has provided detailed reports on various factors such as the incidence, DALYs (Disability-Adjusted Life Years), deaths, and major risk factors of intracerebral hemorrhage in different regions worldwide over time. Therefore, in this study, based on the latest GBD study we provided comparable and comprehensive information on the global burden of intracerebral hemorrhage and its attributable risk factors from 1990 to 2019.

## Methods

We defined stroke according to WHO criteria, involving the rapid development of clinical signs, typically focal disturbances of cerebral function 40 lasting >24 h or resulting in death. Intracerebral hemorrhage was characterized by a focal collection of blood in the brain unrelated to trauma. The definition is derived from https://www.healthdata.org/research-analysis/diseases-injuries/factsheets. The code 161 in ICD-10 specifically refers to nontraumatic intracerebral hemorrhage according to The International Classification of Diseases and Injuries (ICD-10). In our study, we reported the incidence, death, and DALYs of intracerebral hemorrhage by age, risk factor, and temporal trends spanning from 1990 to 2019. The GBD study examined these factors across various demographic categories, including age, sex, and risk factors of intracerebral hemorrhage [13]. Additionally, we conducted a comprehensive analysis of temporal trends spanning from 1990 to 2019. Our study aligns with the methodologies employed by the GBD study 2019, which used a rigorous and standardized set of analytical procedures for estimating intracerebral hemorrhage incidence, death, and DALYs [14]. In simple terms, the incidence of intracerebral hemorrhage is calculated as the number of new cases per 100,000 population per year. Age-standardized using the direct method of the reference population to allow comparability between regions with different age structures. Mortality rates are determined by calculating the number of deaths per 100,000 population, again age-standardized. This method is adjusted for changes in age distribution across countries and regions. DALYs are estimated by summing two components: 1. YLL (Years of Life Lost): Calculated by subtracting the age at death from the standardized life expectancy, reflecting premature mortality; 2. YLD (Years lived with disability): Estimated by multiplying the prevalence of cerebral hemorrhage by a disability weight factor specific to that condition.

The DisMod-MR 2.1, developed by the IHME (Institute for Health Metrics and Evaluation) in Seattle, Washington, United States, is a Bayesian mixed-effects meta-regression tool and it was employed to model and generate estimates of the disease burden across diverse conditions in 204 countries and territories in all regions of the world spanning the period from 1990 to 2019 [14, 15]. This sophisticated tool allowed us to adjust and model our data effectively [14]. It is essential to recognize that individuals living with intracerebral hemorrhage often face both short-term disability and enduring health challenges. Besides, DALYs serve as a valuable metric for quantifying the combined short- and long-term burden of intracerebral hemorrhage [16]. By adopting these rigorous analytical methods and comprehensive data sources, our study provides a robust and detailed assessment of the impact of intracerebral hemorrhage, enabling us to better understand its incidence, and the associated disability burden over time.

We reported the number and age-standardized rate of incidence, death, and DALYs, along with 95% UI (uncertainty intervals), stratified by age and sex in order to describe the global epidemiology and burden of intracerebral hemorrhage [14]. Numbers and rates of incidence, death, and DALYs of six primary causes of intracerebral hemorrhage from 1990 to 2019 worldwide were categorized and analyzed by age. We extrapolated the age structures of patients with intracerebral hemorrhage and calculated the average age of onset. In addition, the evaluation of the burden of intracerebral hemorrhage took into account the backdrop of the national-level development, as constrained by SDI. This index is a comprehensive measure that incorporates three key components: lag-distributed *per capita* income, the average educational attainment of population aged 15 and older, and the total fertility rate for population under the age of 25 [17]. To investigate the relationship between SDI and the burden of disease in cerebral hemorrhage, we used Spearman’s Rho correlation analysis to determine the correlation between ASIR, ASMR, and ASDR and sociodemographic factors in cerebral hemorrhage. In our analysis, we considered a correlation to be weak if the absolute value of rho was between 0.1 and 0.3, we categorized it as moderate if rho was between 0.3 and 0.5, and we categorized it as strong if rho was between 0.5 and 0.6 [14].

### Bayesian Age-Period-Cohort Analysis

Using GBD data from 1990 to 2019, we set out to forecast the burden of disease from 2020 to 2030. Our methodology comprised two main steps:First, we collected data on the incidence and mortality rates of intracerebral hemorrhage across all age groups (segmented in 5-year intervals) globally and regionally from 1990 to 2019. Then, we applied a specific formula — dividing the incidence (or mortality) cases within the same age group by the corresponding rate to recalculate the corresponding annual total populations [18]. Based on this, we utilized the BAPC model to predict the disease burden from 2020 to 2030. The Age-Period-Cohort (APC) models analyze registry data by considering the individual’s age group, the period of the event, and the individual’s birth cohort [19]. BAPC models are distinctive in their approach as they do not rely on parametric assumptions. Bray’s work, which involved comparing the predictions of the linear power model with those of the classical model and the Bayesian APC model, led to the conclusion that Bayesian APC models offer more logical and reliable forecasts [20, 21].

We conducted all statistical analyses and visualizations using R statistical software (version 4.2.3). Statistical significance was defined as a p-value <0.05. Since this study relies on publicly available datasets, no ethical review was required.

## Results

### Global Burden and Temporal Trend in Intracerebral Hemorrhage

**FIGURE 1:**
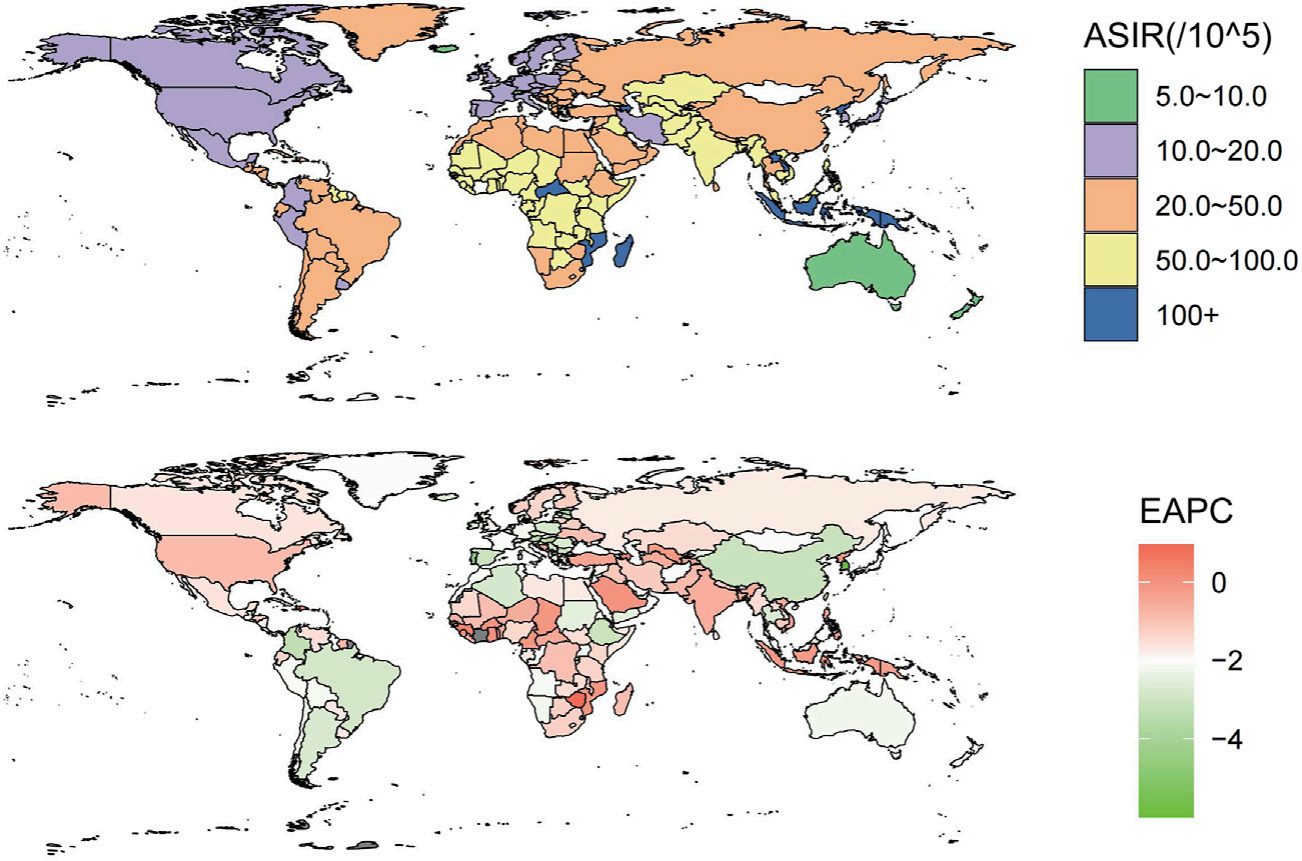
These maps show the age-standardized incidence rate of intracerebral hemorrhage per 100,000 people (Global, 2019) and the estimated annual percentage change for both sexes (Global, 1990–2019).

In 2019, the incident cases of intracerebral hemorrhage were 3.41 million (95% UI 2.97 to 3,91) people worldwide, including 1.83 million (95% UI 1.08–1.44) males and 1.58 (95% UI 1.38–1.81) females, an increase of 43.2% from 2.38 million (95% UI 2.06–27.5) in 1990. However, the ASIR of intracerebral hemorrhage in 2019 was 41.81 per 100,000 (95% UI 36.53–47.88), with more males [47.17 per 100,000 (95% UI 41.35–53.91)] than females [36.81 per 100,000 (95% UI 32.16–42.21)], and from 1990 to 2019, the ASIR decreased [−1.52% (95% UI −1.69 to −1.34)]. The decline was greater for females than for males. The ASIR of all regions also shrunk (estimated annual percentage change <0) (Supplementary Table S1; Figure 1). The greatest rises in incident cases were seen in Oceania (121.5%) and the biggest drop was observed in Central Europe (−30.5%). The incident number increased in the vast majority of areas between 1990 and 2019 except for central Europe, eastern Europe, high-income Asia Pacific, southern Latin America, and Western Europe, and we can see that most of them were in developed regions (Supplementary Table S1). Globally, the number of intracerebral hemorrhage deaths in 2019 was 2.89 million (95% UI 2.64–3.10), an increase of 37.5% compared with 1990 (2.10 million 95% UI 1.93–2.32). The ASMR of intracerebral hemorrhage in 2019 was [36.04 per 100,000(95% UI 32.98–38.67)], with the greatest decrease occurring in High-income Asia Pacific [−4.07% (95% UI −4.25 to −3.9)] (Supplementary Table S3). In addition, intracerebral hemorrhage contributed to 6.86 million (95% UI 6.33–7.37) DALYs cases globally in 2019, a rise of 25.3% compared with 1990. Despite the surges, from 1990 to 2019, the ASDR declined [estimated annual percentage change −1.64% (95% CI −1.82 to −1.46)], with the biggest reduction likewise in high-income Asia Pacific [−3.99% (95% UI −4.14 to −3.84)] (Supplementary Table S4).

### Variation in Intracerebral Hemorrhage Burden at Country Level

At the country level, in 2019, Mongolia had the global highest ASIR [166.54 per 100,000 people (95% UI 156.48–179.06)], while Switzerland had the lowest [8.11 per 100,000 people (95% UI 7.12–9.12)]. The global highest ASDR [5,250.9 per 100,000 people (95% UI 4266.16–6,444.23)] and ASMR [214.62 per 100,000 people (95% UI 177.64–255.29)] of intracerebral hemorrhage were observed in Solomon Islands, whereas the lowest ASDR [87.71 per 100,000 people (77.31–99.04)] and ASMR [5.01 per 100,000 people (4.24–5.82)] were seen in Switzerland (Supplementary Figure S1; Supplementary Table S2). From 1990 to 2019, the country with the largest drop in ASIR [-0.53% (95% UI −0.56 to −0.5)], ASMR [-0.87% (95% UI −0.89 to −0.82)], and ASDR [-0.87% (95% UI −0.89 to −0.82)] was Republic of Korea. Saint Vincent and the Grenadines had the highest increase in ASIR [0.42% (95% UI 0.34–0.5)], while the Philippines showed the largest increase in ASMR and ASDR [0.37 (95% UI 0.04–0.69)] and [0.6 (95% UI 0.1–0.98)] (Supplementary Figure S1; Supplementary Table S2). China notably had the highest number of global incident cases [0.85 million (95% UI 0.70–1.01)], death cases [1.07 million (95% UI 0.92–1.24)], and DALYs [22.21 million (95% UI 18.99–25.78)] due to intracerebral hemorrhage in 2019.

### Variation in 2019 in Intracerebral Hemorrhage Burden by Sex and Age Group

**FIGURE 2:**
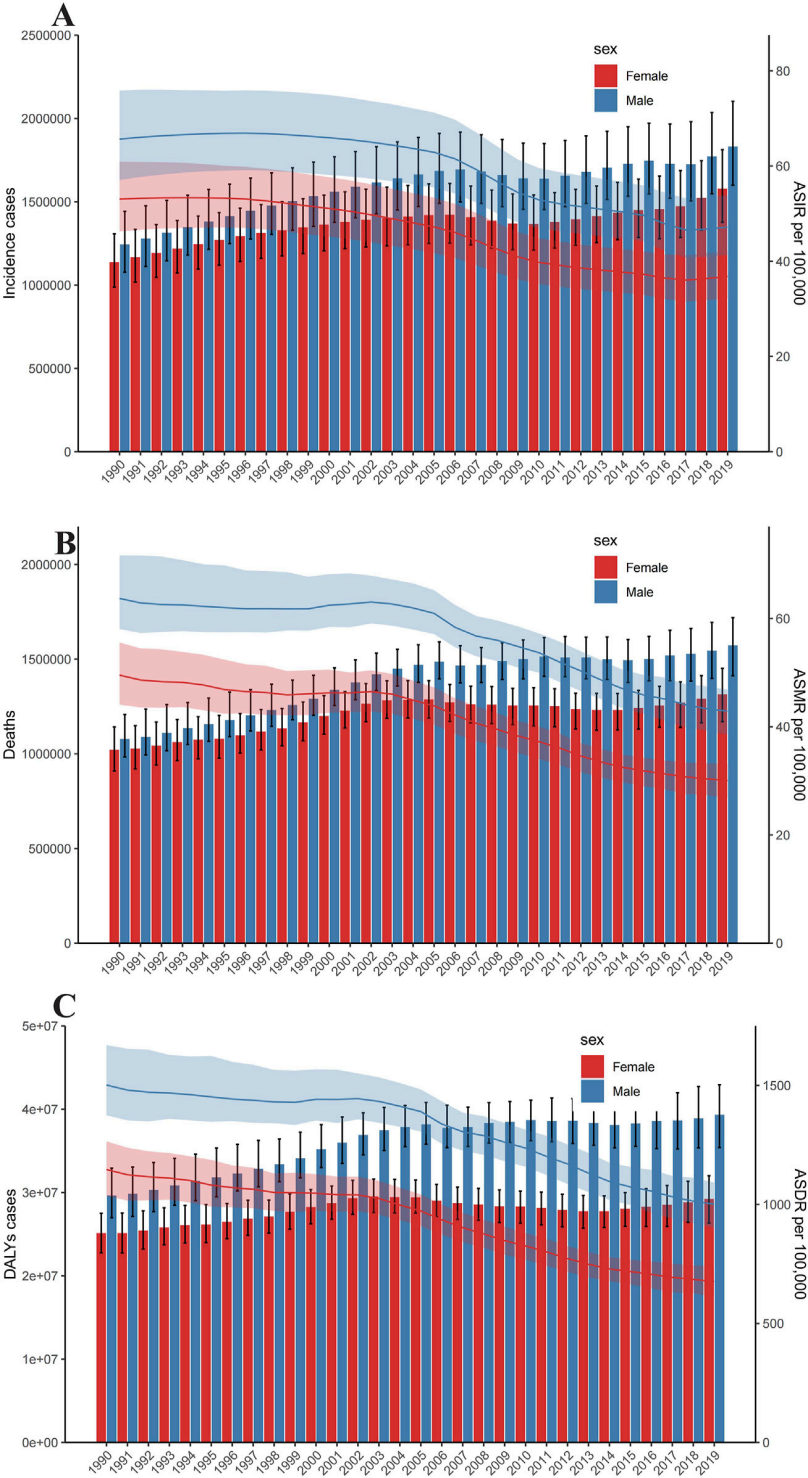
Trends in numbers and age-standardized incidence rate of incident cases **(A)**, deaths **(B)**, and disability-adjusted life years cases **(C)** of Intracerebral hemorrhage. Error bars indicate the 95% uncertainty interval (UI) for numbers. Shading indicates the 95% UI for rates (Global, 1990–2019).

**FIGURE 3:**
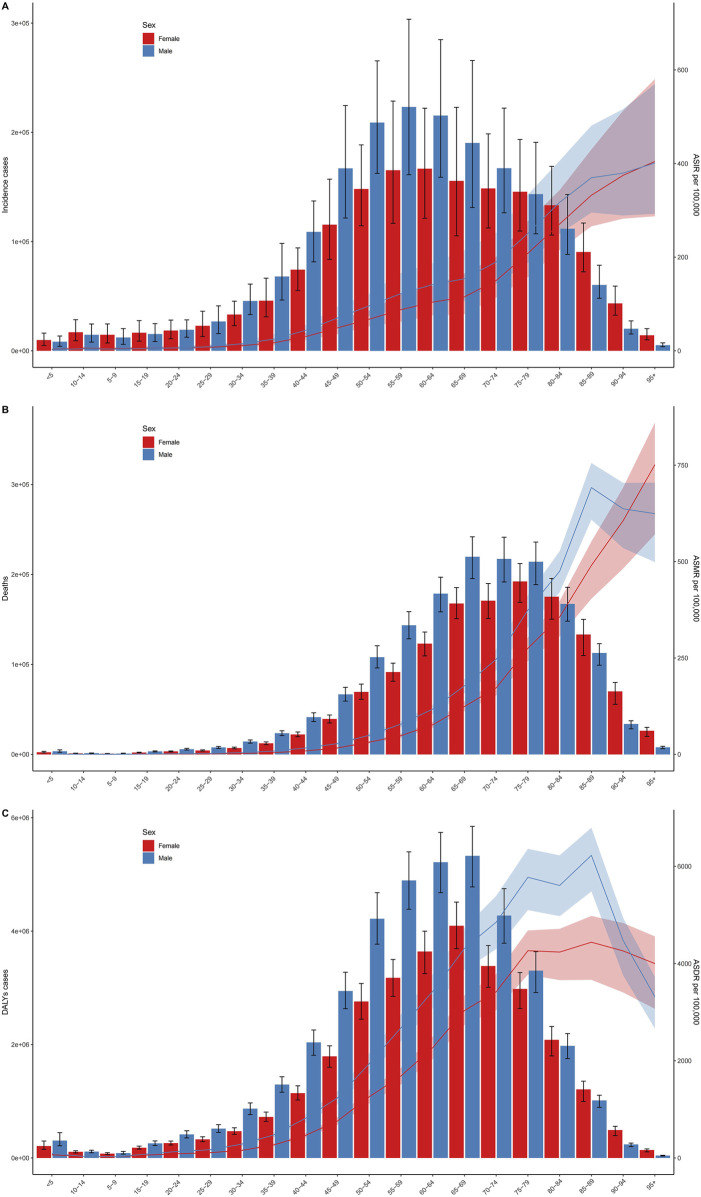
Age-specific numbers and rates of incident cases **(A)**, deaths **(B)**, and disability-adjusted life years cases **(C)** of Intracerebral hemorrhage by sex. Error bars indicate the 95% uncertainty interval (UI) for numbers. Shading indicates the 95% UI for rates (Global, 2019).

The incidence of intracerebral hemorrhage was higher in males than in females, with a correspondingly elevated age-standardized incidence rate in males (Figure 2A). Similarly, the number of deaths from intracerebral hemorrhage was higher in males than in females, and the age-standardized mortality rate was also higher in males (Figure 2B). In addition, as for DALYs, the cases and the age-standardized rate for intracerebral hemorrhage were both higher in men than women (Figure 2C). The trends observed in 2019 for incidence cases, age-standardized rates, DALYs, and deaths were consistent across all years. The cases of incident, DALYs, and deaths all increased annually, while the age-standardized rate fell first and then rose (Figure 2). Figure 3 displays age-specific incidence cases and rates, DALYs, and mortality for intracerebral hemorrhage categorized by sex. The incident cases of intracerebral hemorrhage for males peaked between 55–59 years but the largest incident case in females was between 60–64 years (Figure 3A). Besides, the number of DALYs due to intracerebral hemorrhage reached its peak between 65–69 years in both males and females, with a higher number in males than in females within this age range (Figure 3C). The number of deaths due to intracerebral hemorrhage peaked between 65–69 years in males, while in females, it occurred between 75–79 years. Females in this age group experienced more deaths than males after the ages of 75–79 years (Figure 3B). The incident and mortality rate rised with age for both sexes. Nevertheless, the DALYs rate subsided after 75–79 years in both sexes.

### Variation in Intracerebral Hemorrhage Burden by SDI

**FIGURE 4:**
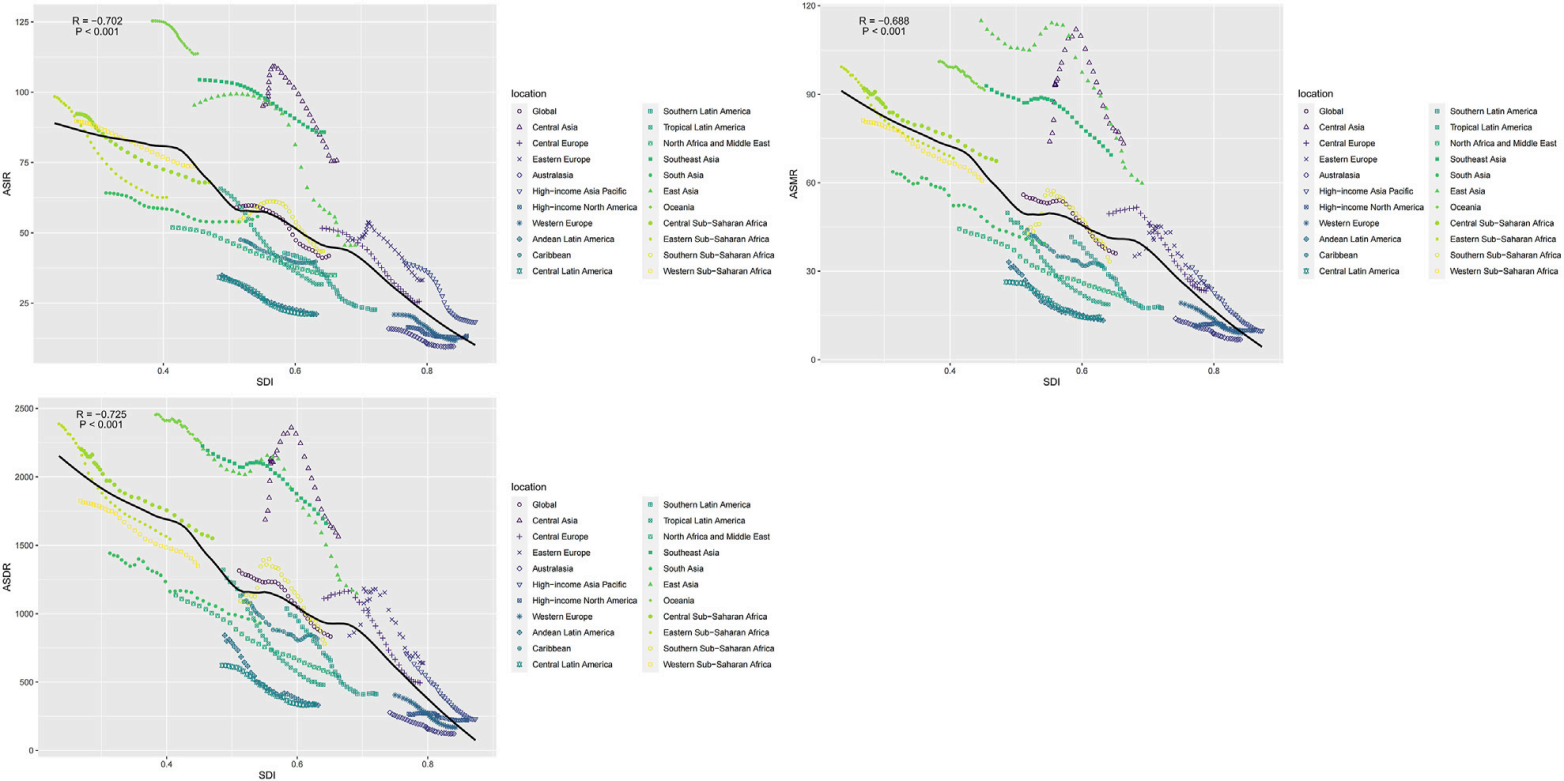
The correlation between global age-standardized incidence rate, mortality rate, and disability-adjusted life year rate and the socio-demographic index for intracerebral hemorrhage for both sexes in different regions (Global, 1990–2019).

Countries with higher SDI exhibited have higher incidence rates than did those with lower SDI (Figure 4). Spearman’s rho correlation tests indicated a robust negative correlation between ASIR (rho = −0.702; p < 0.001), ASMR (rho = −0.688; p < 0.001) and ASDR (rho = −0.725; p < 0.001) and SDI. From 1990 to 2019, the ASIR, ASMR and ASDR showed similar trends with an increase in sociodemographic index. At theregional level, the ASIR, ASMR and ASDR of intracerebral hemorrhage also displayed a decreasing trend with an increase in SDI during 1990–2019. In southeast Asia, central Asia, east Asia, and Oceania, the observed intracerebral hemorrhage burden estimates exceeded the expected level based on the SDI during 1990–2019. Central Asia stood out, particularly with SDI peaking at approximately 0.56 and subsequently experiencing a rapid decline.

### Risk Factors Associated With Death Cases and ASMR of Intracerebral Hemorrhage

**FIGURE 5:**
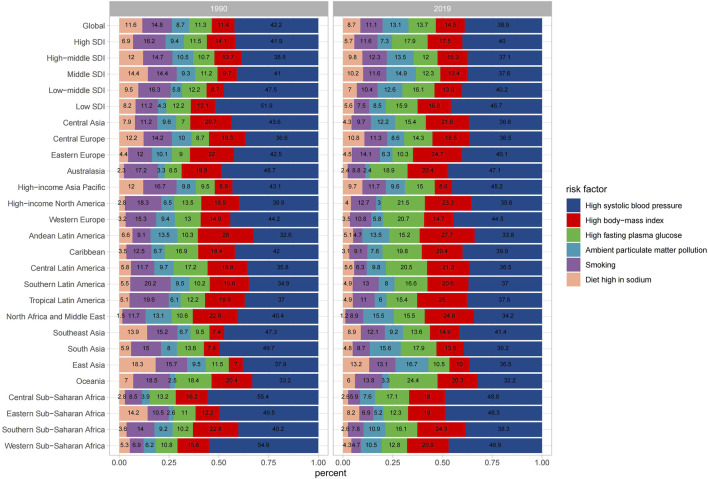
Percentage contributions of major risk factors to age-standardized death for intracerebral hemorrhage (Global, 1990 and 2019).

Of all the potential risk factors reported in GBD 2019, intracerebral hemorrhage deaths worldwide were primarily attributed to several factors, including high systolic blood pressure, high body−mass index, high fasting plasma glucose, ambient particulate matter pollution, smoking, and diet high in sodium. Figure 5 illustrates the annual percentage contribution of these risk factors to death cases. In 1990, worldwide intracerebral hemorrhage deaths were predominantly attributed to high systolic blood pressure, followed by smoking and a diet high in sodium (Figure 5). However, by 2019, the proportion of deaths attributed to smoking and diet high in sodium had declined, with high body−mass index and high fasting plasma glucose ranked in second and third positions as primary risk factors. This shift likely reflected changes in people’s lifestyles over time. Both in 2019 and 1990, regions with a high SDI consistently exhibited a higher prevalence of high body mass index and fasting plasma glucose compared to other SDI regions. In middle SDI regions, the proportion of deaths due to a high-sodium diet was higher compared to other regions. The trend in ASMR attributed to high systolic blood pressure was consistently the highest for both males and females from 1990 to 2019 (Supplementary Figure S2). However, for males, the ASMR trend caused by smoking was significantly higher than that for females. High fasting plasma glucose related ASMR exhibited a pattern of initially rising and then steadily declining over time. The ASDR exhibited a similar trend to that of ASMR (Supplementary Figure S3).

### Intracerebral Hemorrhage Incidence and Death Projections Up to 2030

**FIGURE 6:**
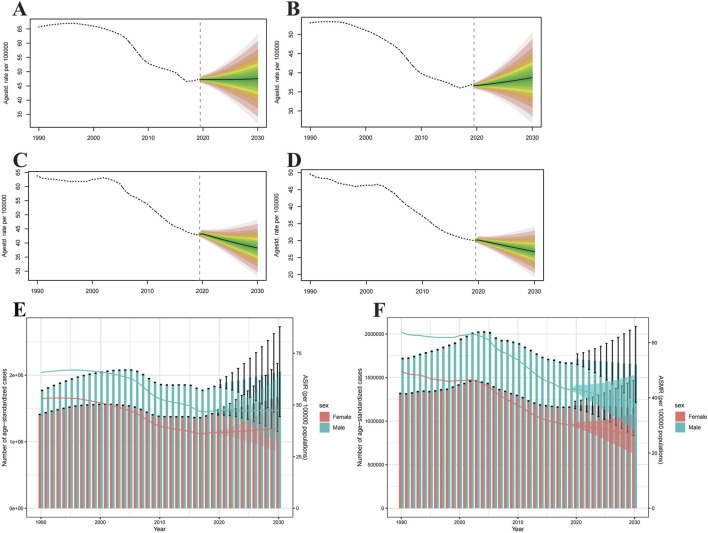
Projections of age-standardized incidence rate **(A,B)** and age-standardized mortality rate **(C,D)** in males and females from 2020 to 2030. The open dot represents the observed value, and the fan represents the predicted distribution between the 2.5% and 97.5% quantiles. The forecast average is shown as a solid line. The vertical dotted line indicates where the prediction begins. The projections of incidence **(E)** and deaths **(F)** by sexes of colorectal cancer due to intracerebral hemorrhage from 2020 to 2030. The error bar denotes the 95% credible interval of the predictive value (Global).

Globally, ASIR will rise for both sexes by 2030, and more markedly for males (Figures 6A,B). But ASMR is the opposite, it will subside for both sexes (Figures 6C,D). From 2020 to 2030, the age-specific incidence rates for males will increase in most age groups, in addition to those over 80 years of age (Supplementary Figure S4). As for females, the age-specific incidence rates will rise in all age groups (Supplementary Figure S5). From 2020 to 2030, the incidence cases will grow annually, with male incidence cases significantly surpassing that of females, but the number of deaths will decline year by year (Figures 6E,F). For the age-specific mortality rates, both males and females will fall in all age groups (Supplementary Figures S6, S7).

## Discussion

Intracerebral hemorrhage is the second most common subtype of stroke, after ischemic stroke and accounts for approximately 10%–20% of all stroke cases [22]. Our study underscores that intracerebral hemorrhage remains a significant global health concern, with over 3.4 million incident cases reported in 2019. This alarming number highlights the substantial burden this condition poses. It is worth noting that despite a decline in age-standardized incidence rates of intracerebral hemorrhage from 1990 to 2019, the absolute number of cases has surged by nearly 1.5 times. This increase can be attributed to factors such as population growth, aging, advances in diagnostic techniques, and increased awareness of intracerebral hemorrhage. Furthermore, there has been a significant rise in both the absolute number of deaths and the Disability-Adjusted Life Years (DALYs) associated with intracerebral hemorrhage. This trend may be partially attributed to undiagnosed asymptomatic or paroxysmal intracerebral hemorrhages and the lack of standardized treatments for the condition. Notably, our research highlights that the increase in DALY cases and deaths related to intracerebral hemorrhage has surpassed the rise in incident cases. In addition, the incidence rate, and cases of intracerebral hemorrhage in both sexes are expected to continue to increase over the next decade, but the number of deaths and mortality rates is expected to decline, possibly due to advances in diagnostic techniques and improvements in treatment. Therefore, there is an urgent need not only for early detection of intracerebral hemorrhage but also for the development of more effective treatment strategies to proactively manage this condition.

Research findings consistently indicate a higher incidence of intracerebral hemorrhage in males compared to females [23]. Furthermore, males bear a greater burden in terms of Disability-Adjusted Life Years (DALYs) and fatalities associated with intracerebral hemorrhage. This gender-based discrepancy can be attributed to elevated rates of smoking and alcohol consumption among males, as well as a heightened susceptibility to risk factors such as hypertension, diabetes, hypercholesterolemia, and coronary artery disease [24]. Among these risk factors, high blood pressure stands out as the most significant contributor to intracerebral hemorrhage [25], corroborating the findings from GBD 2019 data. Importantly, blood pressure is a modifiable risk factor, particularly in low-income settings, as screening tools require minimal equipment but specialized knowledge is scarce [26].

The time trends of age-standardized DALYs and mortality rates for stroke show significant variations across different SDI quintiles. The Social Demographic Index primarily comprises factors such as education levels, *per capita* income, and total fertility rate [27]. Consequently, social factors have a substantial impact on age-standardised DALYs, mortality rates, and the dynamics of stroke. It is essential for each region to formulate appropriate strategies based on its specific circumstances. Regions with higher SDI tend to exhibit higher age-standardised DALYs and mortality rates for stroke, whereas lower SDI regions tend to have lower age-standardised DALYs and mortality rates. This discrepancy may be attributed to differences in treatment strategies, including the management of cerebral edema and the utilization of novel medical and surgical techniques, particularly advanced surgical interventions [28]. These treatment strategies are distributed unevenly across regions with varying socio-economic environments. In recent years, significant progress has been made in reducing the burden of stroke in high SDI regions. It is imperative to actively sustain the significant decline in age-standardized mortality rates and DALY rates in high SDI areas while taking measures to prevent the increase in age-standardized mortality rates in low SDI regions. The future demands increased focus on stroke prevention, diagnosis, and treatment in low SDI regions, and it is time to take action [22].

It is worth highlighting that the incidence of intracerebral hemorrhage peaks in the elderly population. Simultaneously, in the elderly, there is a pronounced increase in intracerebral hemorrhage-related mortality and DALYs. This can be attributed to a higher prevalence of cerebral amyloid angiopathy and hypertension among the elderly, along with increased use of anticoagulant medications in this age group [29]. These findings emphasize the critical need for increased attention to intracerebral hemorrhage management in the elderly population, without neglecting the diagnosis and treatment of intracerebral hemorrhage in younger patients.

However, this review has certain limitations that warrant consideration. Firstly, it relies on data from the Global Burden of Disease (GBD) database, which may be subject to variations in reporting and recording across different countries and regions, potentially affecting data accuracy and completeness [30]. Secondly, the data range from 1990 to 2019, which, while extensive, might not fully capture longer-term trends and influencing factors. Thirdly, this review primarily focuses on epidemiological data related to intracerebral hemorrhage and does not delve into the impact of treatments and interventions on patient outcomes, a crucial aspect in understanding the holistic management of this condition.

### Conclusion

Our research indicates that intracerebral hemorrhage remains a significant public health challenge. Although progress has been made in preventing and treating intracerebral hemorrhage, particularly in areas with higher SDI, its impact is on the rise in regions with lower SDI. Some of the high-risk risk factors for intracerebral hemorrhage are amenable to intervention to some extent, hence, it is imperative to implement more cost-effective strategies and interventions focusing on modifiable risk factors. This is especially crucial in regions facing a high or escalating burden of cerebral hemorrhage.
