# Supplementary figures and images for: Intracerebral Hemorrhage: The Global Differential Burden and Secular Trends From 1990 to 2019 and Its Prediction up to 2030
Source: Int J Public Health. 2025 May 21;70:1607013. doi: 10.3389/ijph.2025.1607013 (PMC12133604; doi:10.3389/ijph.2025.1607013)

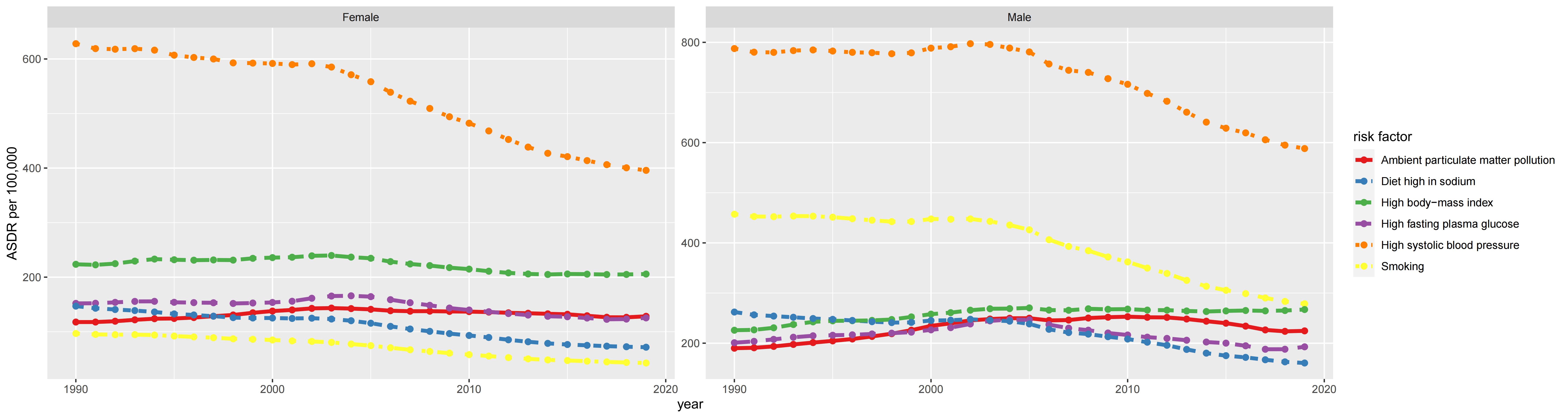

Supplement: Supplementary file 1 [file Image3.jpeg]

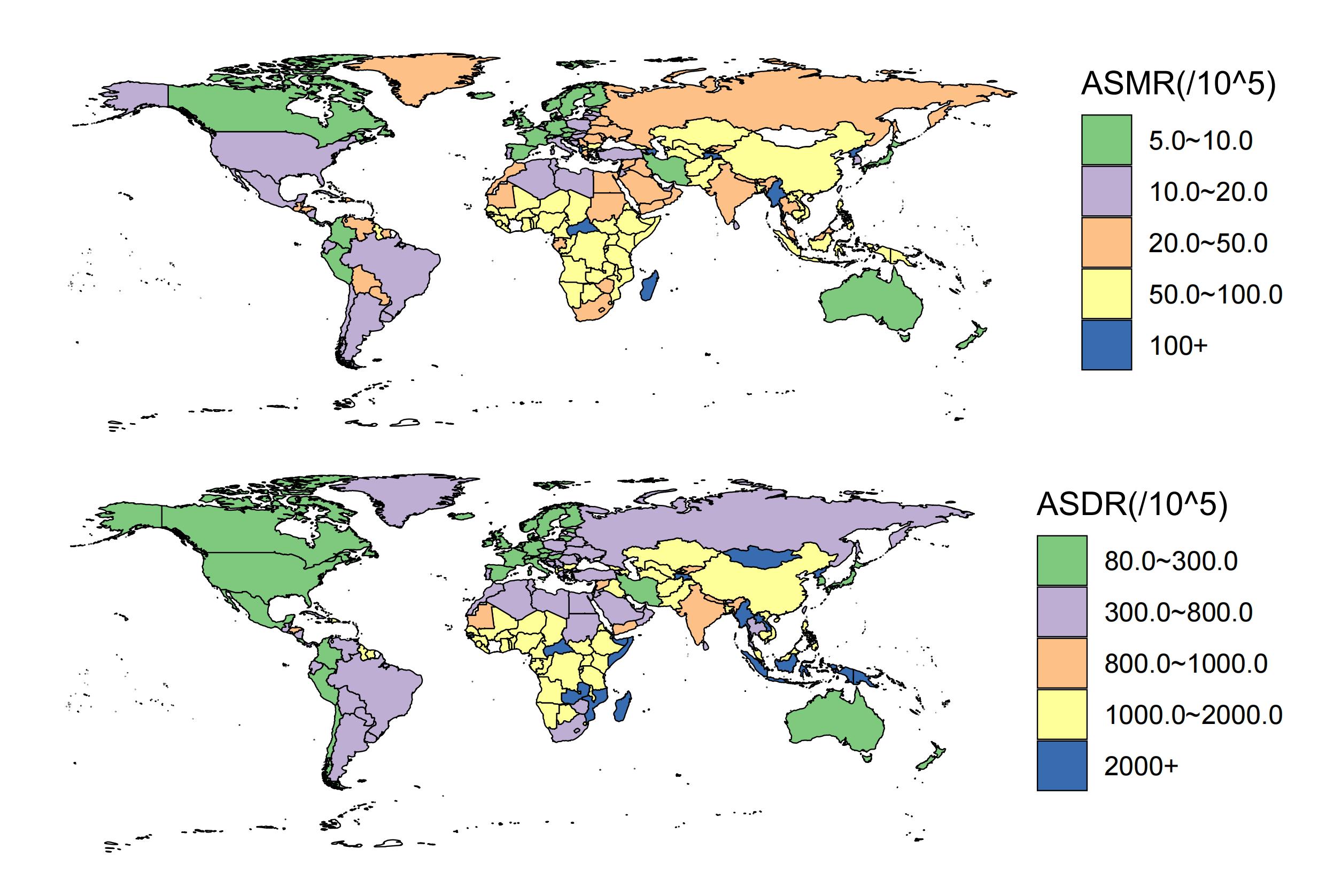

Supplement: Supplementary file 3 [file Image1.jpeg]

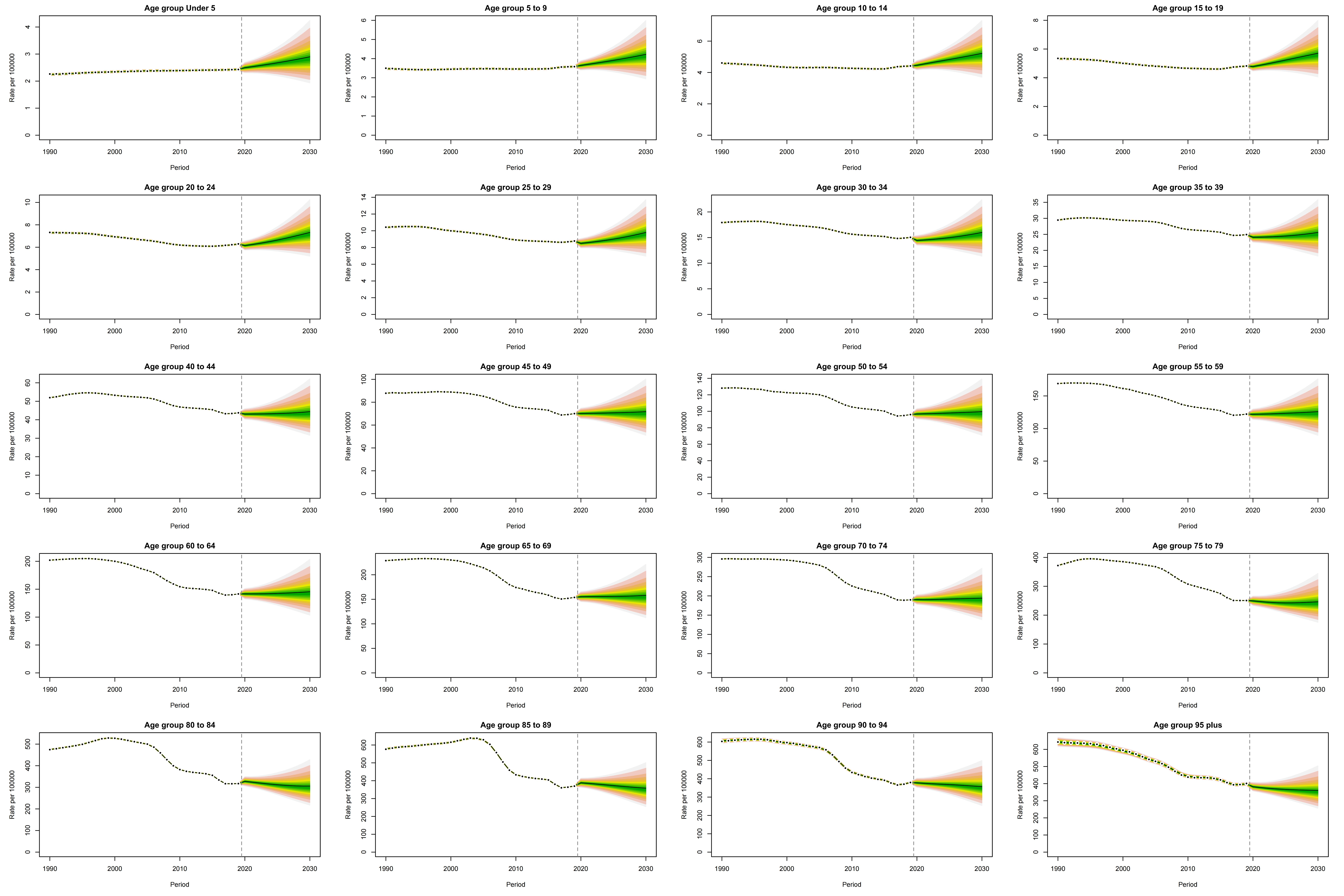

Supplement: Supplementary file 4 [file Image4.jpeg]

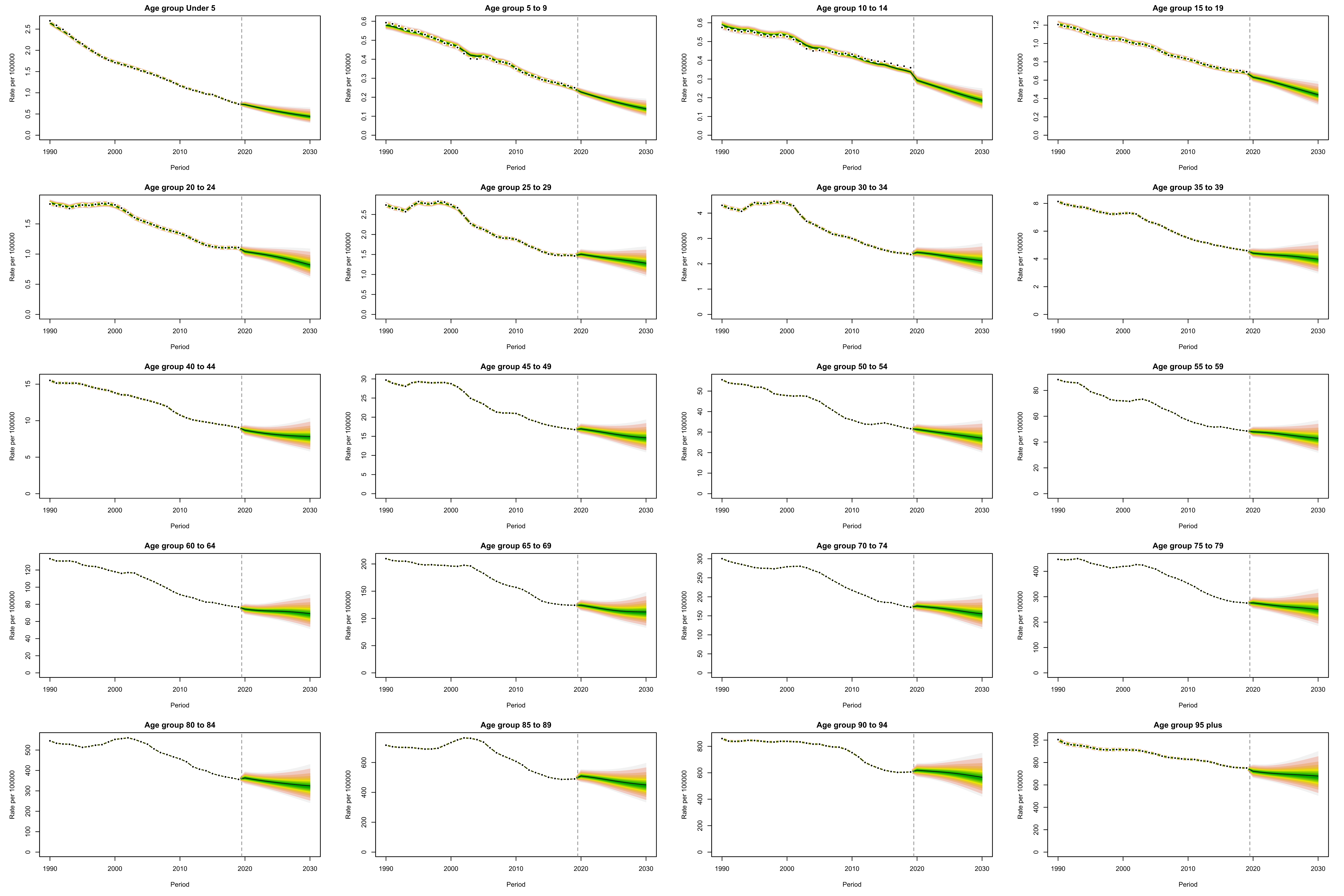

Supplement: Supplementary file 5 [file Image7.jpeg]

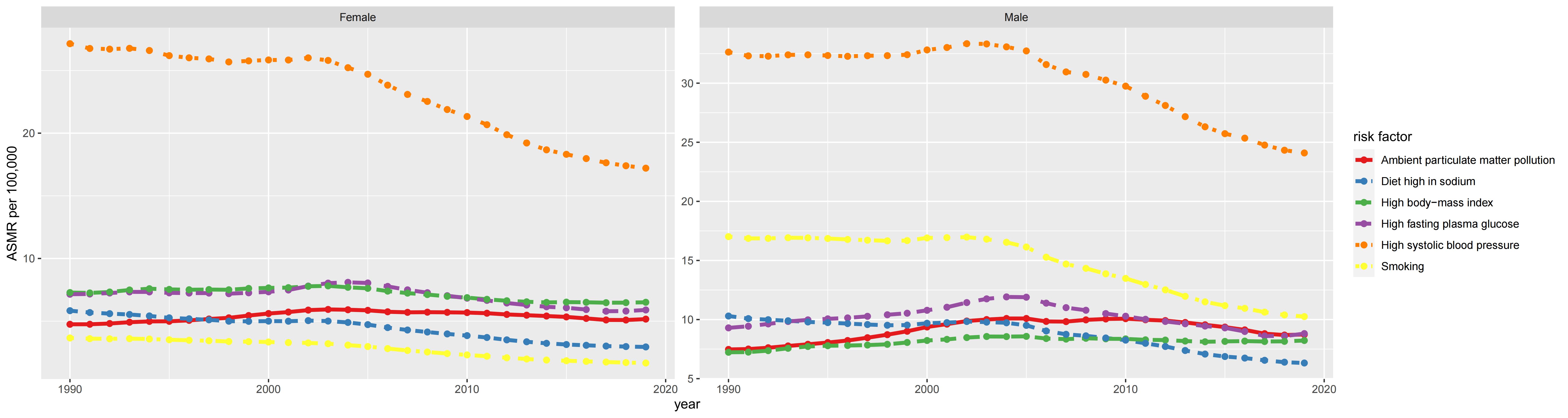

Supplement: Supplementary file 6 [file Image2.jpeg]

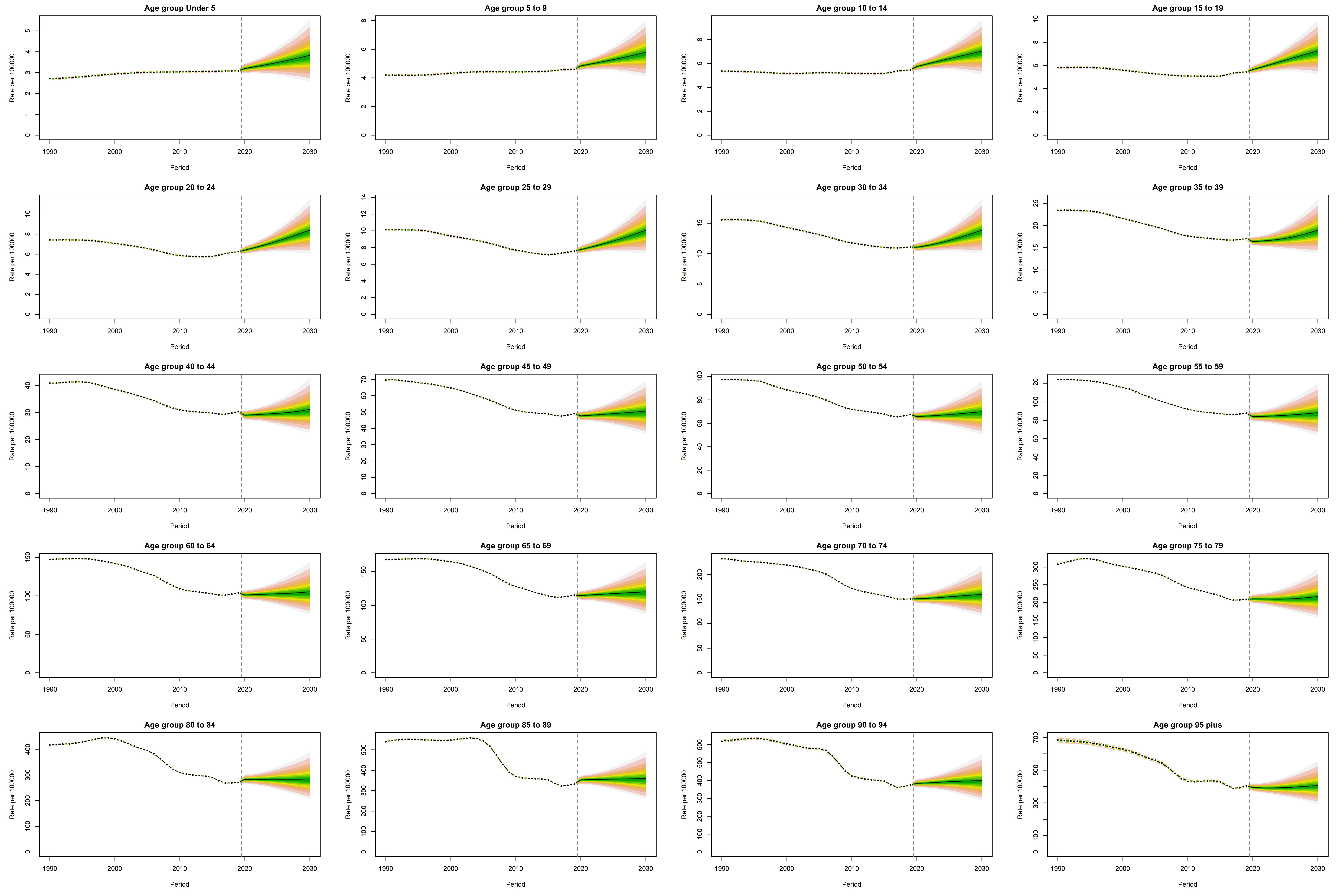

Supplement: Supplementary file 7 [file Image5.jpeg]

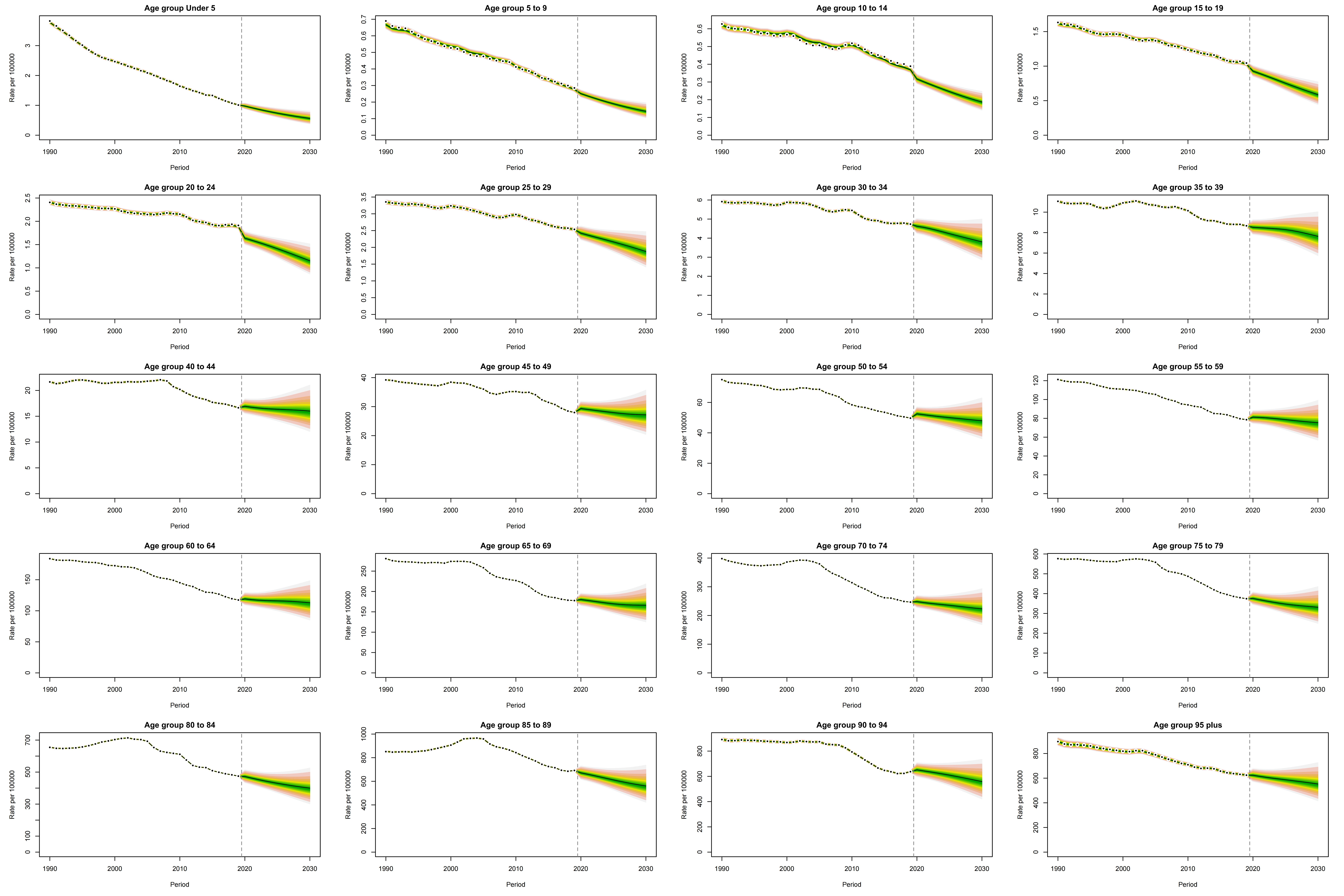

Supplement: Supplementary file 11 [file Image6.jpeg]
