# Supplementary material for: Intracerebral Hemorrhage: The Global Differential Burden and Secular Trends From 1990 to 2019 and Its Prediction up to 2030
Source: Int J Public Health. 2025 May 21;70:1607013. doi: 10.3389/ijph.2025.1607013 (PMC12133604; doi:10.3389/ijph.2025.1607013)
Supplement: Supplementary file 2 [file Table1.docx]

Supplementary Table S1: Incident number, age standardized incident rate in 1990 and 2019 the percentage change of incident number between 1990 and 2019 for intracerebral hemorrhage with EAPC.

| location | Incident number  in 1990 | Incident number  in 2019 | ASIR  in 2019 | EAPC  1990 to 2019 | Change of incident number between 1990 and 2019 |
| --- | --- | --- | --- | --- | --- |
| Global  Both sexes | 2381001 (2060224 to 2751095) | 3409122 (2970474 to 3909194) | 41.81 (36.53 to 47.88) | -1.52% (-1.69 to -1.34) | 43.2% |
| Male | 1243369 (1077442 to 1442506) | 1830930 (1599645 to 2102661) | 47.17 (41.35 to 53.91) | -1.43% (-1.62 to -1.23) | 47.3% |
| Female | 1137632 (988021 to 1307257) | 1578192 (1377250 to 1812731) | 36.81 (32.16 to 42.21) | -1.64% (-1.8 to -1.48) | 38.7% |
| Region  Australasia | 3585 (3238 to 3951) | 4511 (4024 to 5056) | 9.6 (8.59 to 10.75) | -2.15% (-2.35 to -1.96) | 25.8% |
| Caribbean | 13000 (12058 to 14102) | 20369 (18910 to 22016) | 39.79 (36.94 to 43.1) | -0.75% (-0.82 to -0.67) | 56.7% |
| Central Asia | 45115 (41973 to 48893) | 55139 (51619 to 59536) | 75.74 (71.11 to 81.44) | -0.99% (-1.32 to -0.65) | 22.2% |
| Central Europe | 72177 (66986 to 78368) | 50169 (46190 to 54479) | 25.62 (23.62 to 27.86) | -2.88% (-3.05 to -2.71) | -30.5% |
| Central Latin America | 33443 (30010 to 37657) | 50913 (45293 to 57528) | 21.24 (18.95 to 23.89) | -1.96% (-2.09 to -1.82) | 52.2% |
| Central Sub-Saharan Africa | 23234 (21193 to 25383) | 41167 (37433 to 45206) | 68.06 (62.37 to 74.98) | -1.25% (-1.32 to -1.17) | 77.2% |
| East Asia | 751855 (615481 to 908112) | 895820 (750140 to 1061517) | 45.53 (38.57 to 53.39) | -2.99% (-3.48 to -2.5) | 19.1% |
| Eastern Europe | 126594 (107415 to 149350) | 103769 (88158 to 121871) | 33.2 (28.58 to 38.6) | -1.57% (-1.93 to -1.2) | -18% |
| Eastern Sub-Saharan Africa | 83459 (75095 to 92940) | 118819 (106849 to 132361) | 62.64 (56.46 to 69.7) | -1.84% (-1.93 to -1.75) | 42.4% |
| Andean Latin America | 8836 (8026 to 9768) | 12355 (11181 to 13723) | 21.1 (19.11 to 23.36) | -1.89% (-1.96 to -1.82) | 39.8% |
| High-income Asia Pacific | 77156 (66051 to 89431) | 67322 (58750 to 77001) | 18.33 (15.97 to 21) | -3.19% (-3.48 to -2.89) | -12.7% |
| High-income North America | 56276 (47051 to 67046) | 78270 (65082 to 93140) | 13.38 (11.26 to 15.91) | -0.93% (-1.1 to -0.77) | 39.1% |
| North Africa and Middle East | 98888 (90031 to 109014) | 163240 (149166 to 179814) | 34.97 (31.83 to 38.62) | -1.59% (-1.66 to -1.52) | 65.1% |
| Oceania | 4483 (4129 to 4882) | 9931 (9106 to 10761) | 113.71 (105.03 to 123.29) | -0.39% (-0.42 to -0.35) | 121.5% |
| South Asia | 389798 (330322 to 457805) | 815704 (692848 to 959240) | 55.8 (47.47 to 65.57) | -0.69% (-0.77 to -0.61) | 109.3% |
| Southeast Asia | 291927 (260237 to 327838) | 545387 (481347 to 615944) | 85.8 (76.38 to 96.92) | -0.77% (-0.83 to -0.71) | 86.8% |
| Southern Latin America | 19647 (18244 to 21448) | 18105 (16700 to 19746) | 22.73 (20.93 to 24.93) | -2.57% (-2.7 to -2.44) | -7.8% |
| Southern Sub-Saharan Africa | 16598 (14471 to 19027) | 25342 (21821 to 29309) | 43.48 (37.51 to 50.47) | -0.99% (-1.32 to -0.66) | 52.7% |
| Tropical Latin America | 68809 (58422 to 80522) | 76852 (64825 to 91015) | 31.72 (26.77 to 37.37) | -2.81% (-2.89 to -2.72) | 11.7% |
| Western Europe | 114754 (103476 to 126865) | 102863 (92103 to 115215) | 11.79 (10.53 to 13.24) | -2.33% (-2.44 to -2.22) | -10.4% |
| Western Sub-Saharan Africa | 81369 (72434 to 91275) | 153074 (137381 to 171048) | 73.66 (66.01 to 82.36) | -0.79% (-0.84 to -0.73) | 88.1% |
