# Supplementary material for: Intracerebral Hemorrhage: The Global Differential Burden and Secular Trends From 1990 to 2019 and Its Prediction up to 2030
Source: Int J Public Health. 2025 May 21;70:1607013. doi: 10.3389/ijph.2025.1607013 (PMC12133604; doi:10.3389/ijph.2025.1607013)
Supplement: Supplementary file 8 [file Table2.docx]

Supplementary Table S2: Incident, death, and DALY numbers; ASIR, ASMR, and ASDR in 2019, and the percentage change of ASIR, ASMR, and ASDR intracerebral hemorrhage between 1990 and 2019, by country.

| location | Incidence | | |  | Deaths | | |  | DALYs | | |
| --- | --- | --- | --- | --- | --- | --- | --- | --- | --- | --- | --- |
|  | 2019 counts | 2019 ASIR per 100,000 | Percentage change in ASIR,1990-2019 |  | 2019 counts | 2019 ASMR per 100,000 | Percentage change in ASMR,1990-2019 |  | 2019 counts | 2019 ASDR per 100,000 | Percentage change in ASDR,1990-2019 |
| Afghanistan | 11653.34 (10651.24 to 12822.64) | 60.97 (55.99 to 66.27) | -0.38 (-0.4 to -0.35) |  | 6960.67 (4904.37 to 9333.13) | 54.05 (36.59 to 72.38) | -0.47 (-0.6 to -0.33) |  | 241903.06 (176657.63 to 324979.11) | 1397.66 (990.95 to 1864.5) | -0.46 (-0.59 to -0.3) |
| Albania | 2921.77 (2697.23 to 3168.35) | 72.25 (66.91 to 78.08) | -0.26 (-0.3 to -0.23) |  | 4142.95 (3233.44 to 5166.27) | 100.64 (79 to 125.33) | -0.25 (-0.41 to -0.05) |  | 62919.75 (48453.56 to 79015.08) | 1529.17 (1184.61 to 1915.41) | -0.32 (-0.47 to -0.14) |
| Algeria | 9769.41 (8701.17 to 10901.08) | 29.08 (25.84 to 32.54) | -0.5 (-0.53 to -0.47) |  | 5275.74 (4128.53 to 6724.99) | 18.51 (14.54 to 23.57) | -0.69 (-0.77 to -0.6) |  | 142325.03 (112671.49 to 177629.99) | 403.44 (322.36 to 501.27) | -0.68 (-0.76 to -0.58) |
| American Samoa | 39 (35.22 to 43.26) | 80.94 (73.65 to 89) | -0.15 (-0.19 to -0.1) |  | 25.82 (22.16 to 29.91) | 59.07 (51.15 to 68.11) | -0.3 (-0.41 to -0.15) |  | 693.41 (583.89 to 817.48) | 1408.82 (1196.69 to 1650.2) | -0.28 (-0.4 to -0.14) |
| Andorra | 13.49 (11.94 to 15.27) | 10.03 (8.76 to 11.42) | -0.33 (-0.37 to -0.29) |  | 8.46 (5.99 to 11.64) | 5.67 (3.97 to 7.89) | -0.34 (-0.55 to -0.08) |  | 152.25 (110.58 to 208.7) | 110.89 (80.26 to 151.89) | -0.37 (-0.56 to -0.13) |
| Angola | 8893.95 (7993.92 to 9834.56) | 68.72 (62.28 to 75.6) | -0.4 (-0.44 to -0.37) |  | 6228.75 (4793.69 to 7948.28) | 61.79 (48 to 77.02) | -0.37 (-0.51 to -0.19) |  | 189240.69 (145900.27 to 240562.97) | 1446.96 (1129.72 to 1824.26) | -0.41 (-0.55 to -0.22) |
| Antigua and Barbuda | 30.78 (28.05 to 33.92) | 31.07 (28.38 to 34.19) | -0.34 (-0.38 to -0.3) |  | 28.3 (24.26 to 33.09) | 29.54 (25.42 to 34.38) | -0.42 (-0.51 to -0.31) |  | 650.92 (551.64 to 766.13) | 630.68 (535.74 to 740.28) | -0.45 (-0.54 to -0.35) |
| Argentina | 12262.04 (11304.86 to 13397.28) | 23.65 (21.81 to 25.91) | -0.48 (-0.51 to -0.45) |  | 10764.73 (9751.65 to 11774.71) | 19.88 (18.04 to 21.74) | -0.57 (-0.62 to -0.5) |  | 246057.55 (224983.69 to 268012.94) | 472.09 (430.8 to 514.07) | -0.6 (-0.64 to -0.55) |
| Armenia | 1084.67 (978.18 to 1219.39) | 27.99 (25.3 to 31.32) | -0.49 (-0.53 to -0.46) |  | 729.8 (612.86 to 864.16) | 18.13 (15.25 to 21.42) | -0.53 (-0.63 to -0.39) |  | 15608.66 (13161.22 to 18452.16) | 382.39 (322.64 to 450.7) | -0.52 (-0.63 to -0.4) |
| Australia | 3885.01 (3465.22 to 4371.87) | 9.75 (8.71 to 10.93) | -0.4 (-0.44 to -0.36) |  | 3106.31 (2683.89 to 3454.68) | 6.74 (5.9 to 7.47) | -0.52 (-0.56 to -0.47) |  | 49262.24 (44117.12 to 54076.08) | 120.29 (108.58 to 131.5) | -0.56 (-0.6 to -0.52) |
| Austria | 1919.33 (1728.91 to 2143.87) | 11.71 (10.47 to 13.15) | -0.57 (-0.61 to -0.53) |  | 1279.66 (1100.69 to 1434.45) | 6.28 (5.5 to 7.02) | -0.64 (-0.68 to -0.6) |  | 20893.38 (18830.95 to 22964.36) | 120.22 (108.78 to 131.47) | -0.68 (-0.71 to -0.65) |
| Azerbaijan | 8408.96 (7726.25 to 9272.51) | 106.41 (98.32 to 116.19) | 0.06 (0 to 0.12) |  | 6943.11 (5709.01 to 8254.41) | 105.66 (87.38 to 125.11) | 0.29 (0.08 to 0.56) |  | 156725.91 (129463.96 to 185749.55) | 1852.99 (1537.82 to 2187.91) | 0.04 (-0.13 to 0.24) |
| Bahamas | 136.77 (125.91 to 149.33) | 34.1 (31.34 to 37.13) | -0.26 (-0.3 to -0.22) |  | 105.48 (86.27 to 129.82) | 27.84 (22.91 to 34.03) | -0.32 (-0.46 to -0.16) |  | 2820.81 (2287.14 to 3499.09) | 683.27 (557.94 to 842.72) | -0.31 (-0.45 to -0.14) |
| Bahrain | 229.59 (201.74 to 261.22) | 21.82 (19.34 to 24.6) | -0.46 (-0.5 to -0.43) |  | 88.31 (70.37 to 112.65) | 13.05 (10.67 to 16.95) | -0.61 (-0.7 to -0.5) |  | 2951.82 (2393.84 to 3661.65) | 264.62 (217.21 to 326.51) | -0.64 (-0.72 to -0.54) |
| Bangladesh | 87749.37 (79198.63 to 97109.5) | 66.4 (59.99 to 73.33) | -0.11 (-0.16 to -0.07) |  | 82323.1 (58971.14 to 103864.06) | 68.46 (48.81 to 86.03) | -0.33 (-0.5 to -0.15) |  | 1967203.03 (1452603.12 to 2501197.08) | 1477.8 (1103.31 to 1875.47) | -0.37 (-0.53 to -0.2) |
| Barbados | 111.11 (100.61 to 124.11) | 25.33 (22.99 to 28.21) | -0.22 (-0.27 to -0.18) |  | 102.89 (84.78 to 123.99) | 20.95 (17.26 to 25.22) | -0.38 (-0.51 to -0.23) |  | 2213.65 (1807.74 to 2684.3) | 467.75 (381.98 to 568.5) | -0.39 (-0.52 to -0.25) |
| Belarus | 4615.29 (4186.83 to 5154.16) | 31.87 (28.96 to 35.31) | -0.26 (-0.3 to -0.22) |  | 3603.77 (2596.86 to 5030.48) | 22.74 (16.38 to 31.59) | -0.25 (-0.45 to 0.01) |  | 86857.69 (63474.32 to 117432.29) | 573.95 (422.38 to 775.98) | -0.26 (-0.45 to -0.02) |
| Belgium | 2798.15 (2533.58 to 3111.91) | 12.26 (11.1 to 13.64) | -0.42 (-0.45 to -0.38) |  | 2845.97 (2447.54 to 3174.97) | 10.73 (9.41 to 11.86) | -0.46 (-0.53 to -0.41) |  | 43106.73 (38657.91 to 47315.6) | 193.1 (174.8 to 210.61) | -0.54 (-0.58 to -0.49) |
| Belize | 94.53 (85.95 to 105.56) | 30.7 (28.03 to 34.02) | -0.21 (-0.25 to -0.17) |  | 69 (59.95 to 79.51) | 26.01 (22.48 to 29.98) | -0.29 (-0.4 to -0.14) |  | 1829.15 (1582.04 to 2112.41) | 610.75 (530.85 to 705.21) | -0.25 (-0.37 to -0.11) |
| Benin | 4523.02 (4105.94 to 4985.99) | 83.37 (76.05 to 92.3) | -0.12 (-0.16 to -0.08) |  | 3259.6 (2556.2 to 4119.8) | 74.52 (59.86 to 92.15) | -0.18 (-0.35 to 0.03) |  | 89834.29 (67871.97 to 116881.19) | 1611.97 (1252.73 to 2067.53) | -0.21 (-0.38 to 0.02) |
| Bermuda | 17.95 (16.1 to 20.07) | 16.84 (14.96 to 19.06) | -0.39 (-0.44 to -0.35) |  | 11.93 (9.86 to 14.57) | 8.91 (7.39 to 10.85) | -0.61 (-0.7 to -0.5) |  | 238.92 (197.98 to 290.24) | 195.53 (162.59 to 236.98) | -0.62 (-0.7 to -0.52) |
| Bhutan | 234.98 (208.18 to 263.56) | 40.35 (35.71 to 45.39) | -0.38 (-0.42 to -0.34) |  | 158.75 (116.79 to 208.34) | 30.48 (22.53 to 40.02) | -0.45 (-0.6 to -0.21) |  | 3823.26 (2827.78 to 5042.3) | 664.52 (493.35 to 872.19) | -0.47 (-0.62 to -0.25) |
| Bolivia (Plurinational State of) | 2565.61 (2292.28 to 2876.95) | 27.13 (24.42 to 30.2) | -0.42 (-0.45 to -0.38) |  | 2196.79 (1568.4 to 2989.06) | 26.44 (19.04 to 35.75) | -0.49 (-0.61 to -0.33) |  | 55647.14 (39517.06 to 76087.05) | 598.36 (428.39 to 814.63) | -0.53 (-0.65 to -0.38) |
| Bosnia and Herzegovina | 1142.19 (1042.17 to 1248.83) | 21.58 (19.66 to 23.47) | -0.11 (-0.16 to -0.06) |  | 1067.12 (848.27 to 1332.68) | 18.7 (14.87 to 23.41) | -0.24 (-0.41 to -0.01) |  | 20287.99 (16210.16 to 25642.86) | 357.82 (287.54 to 451.5) | -0.34 (-0.48 to -0.14) |
| Botswana | 835.11 (759.12 to 923.03) | 56.23 (51.23 to 62.11) | -0.26 (-0.3 to -0.22) |  | 704.82 (504.71 to 947.17) | 56.93 (41.85 to 75.27) | -0.31 (-0.51 to -0.04) |  | 20117.38 (13980.88 to 27266.79) | 1322.28 (947.24 to 1766.5) | -0.32 (-0.53 to -0.03) |
| Brazil | 74670.98 (62811.59 to 88635.81) | 31.59 (26.57 to 37.33) | -0.52 (-0.54 to -0.5) |  | 43825.95 (40717.26 to 46438.09) | 18.65 (17.26 to 19.79) | -0.63 (-0.65 to -0.6) |  | 1154165.41 (1091357.51 to 1217146.26) | 477.65 (450.93 to 503.79) | -0.64 (-0.67 to -0.62) |
| Brunei Darussalam | 106.43 (94.84 to 120.15) | 33.46 (30.13 to 37.2) | -0.51 (-0.54 to -0.48) |  | 61.61 (53.63 to 71.41) | 28.09 (24.59 to 32.55) | -0.6 (-0.66 to -0.51) |  | 1738.13 (1513.88 to 1998.3) | 573.87 (505.64 to 654.99) | -0.62 (-0.68 to -0.52) |
| Bulgaria | 6261.11 (5838.7 to 6731.21) | 48 (44.98 to 51.65) | -0.52 (-0.55 to -0.5) |  | 7607.9 (6192.61 to 9250.2) | 52.22 (42.37 to 63.72) | -0.52 (-0.61 to -0.4) |  | 147115.96 (118489.5 to 181021.46) | 1101.01 (883.55 to 1361.39) | -0.52 (-0.62 to -0.4) |
| Burkina Faso | 6799.87 (6128.33 to 7518.07) | 67.49 (61.24 to 74.49) | -0.01 (-0.06 to 0.04) |  | 5550.71 (4487.65 to 6758.69) | 66.75 (54.75 to 79.63) | 0.06 (-0.17 to 0.31) |  | 165665.71 (130756.88 to 206271.06) | 1507.73 (1219.02 to 1832.44) | 0.02 (-0.21 to 0.28) |
| Burundi | 3572.07 (3219.25 to 3974.89) | 67.88 (61.58 to 75.07) | -0.49 (-0.52 to -0.46) |  | 3220.01 (2352.46 to 4402.18) | 78.56 (57.44 to 107.01) | -0.42 (-0.57 to -0.23) |  | 93725.03 (69264.36 to 127977.61) | 1796.1 (1324.31 to 2442.73) | -0.47 (-0.62 to -0.27) |
| Cabo Verde | 261.69 (235.04 to 293.33) | 56.66 (50.84 to 63.32) | -0.17 (-0.21 to -0.12) |  | 196.78 (164.13 to 245.72) | 47.24 (39.4 to 58.98) | 0.07 (-0.12 to 0.37) |  | 4495.61 (3718.74 to 5518.41) | 1017.85 (839.72 to 1252.32) | -0.09 (-0.26 to 0.15) |
| Cambodia | 11829.57 (10794.18 to 13018.98) | 97.08 (89.04 to 106.46) | -0.23 (-0.27 to -0.19) |  | 8974.2 (7226.58 to 10782.5) | 84.75 (67.49 to 100.68) | -0.27 (-0.44 to -0.11) |  | 230369.77 (185737.7 to 278810.41) | 1870.64 (1509.15 to 2243.09) | -0.33 (-0.48 to -0.17) |
| Cameroon | 11808.05 (10722.76 to 12976.14) | 88.19 (80.36 to 97.21) | -0.02 (-0.07 to 0.02) |  | 7733.31 (5830.63 to 10247.87) | 72.28 (55.75 to 92.71) | -0.11 (-0.33 to 0.17) |  | 229744.38 (169139.38 to 307772.95) | 1635.96 (1230.52 to 2153.02) | -0.11 (-0.35 to 0.22) |
| Canada | 6517.62 (5810.88 to 7343.89) | 10.16 (9.03 to 11.42) | -0.36 (-0.4 to -0.3) |  | 4609.89 (4028.55 to 5150.73) | 6.23 (5.5 to 6.92) | -0.41 (-0.47 to -0.35) |  | 80313.34 (72669.43 to 88565.6) | 122.34 (110.85 to 134.5) | -0.44 (-0.49 to -0.38) |
| Central African Republic | 2719.8 (2468.09 to 2997.09) | 102.06 (93.18 to 112.27) | -0.1 (-0.14 to -0.06) |  | 2060.17 (1511.81 to 2764.36) | 101.85 (74.02 to 136.35) | -0.17 (-0.36 to 0.08) |  | 65841.57 (48208.29 to 87863.94) | 2526.24 (1863.87 to 3359.94) | -0.18 (-0.37 to 0.08) |
| Chad | 5638.91 (5132.54 to 6212.22) | 88.69 (81.21 to 97.54) | -0.02 (-0.07 to 0.02) |  | 4106.26 (3231.55 to 5222.81) | 77.85 (62.56 to 98.16) | 0 (-0.2 to 0.23) |  | 126202.17 (97206.17 to 160360.82) | 1792.18 (1398.96 to 2277.01) | -0.02 (-0.23 to 0.24) |
| Chile | 4842.75 (4349.69 to 5395.41) | 21.38 (19.23 to 23.85) | -0.47 (-0.5 to -0.42) |  | 3110.47 (2772.34 to 3423.76) | 12.98 (11.58 to 14.28) | -0.62 (-0.67 to -0.57) |  | 69628.19 (63274.46 to 76201.04) | 294.98 (268.54 to 322.56) | -0.64 (-0.67 to -0.6) |
| China | 847223.93 (702065.11 to 1009802.84) | 44.6 (37.48 to 52.68) | -0.53 (-0.56 to -0.5) |  | 1069121.19 (924664.77 to 1236579.14) | 60.09 (52.3 to 68.99) | -0.48 (-0.59 to -0.38) |  | 22210554.93 (18994317.71 to 25780050.72) | 1142.24 (978.07 to 1322.51) | -0.5 (-0.6 to -0.39) |
| Colombia | 8943.73 (8009.04 to 9954.25) | 17.09 (15.25 to 19.06) | -0.56 (-0.59 to -0.52) |  | 5389.58 (4136.03 to 6905.07) | 10 (7.68 to 12.85) | -0.67 (-0.74 to -0.57) |  | 117941.73 (91591.2 to 150687.6) | 224.36 (174.41 to 286.26) | -0.68 (-0.75 to -0.59) |
| Comoros | 306.31 (276.84 to 339.43) | 59.36 (53.85 to 65.59) | -0.46 (-0.48 to -0.43) |  | 253.37 (189.93 to 327.14) | 55.28 (41.54 to 70.92) | -0.39 (-0.54 to -0.03) |  | 6385.44 (4687.84 to 8352.13) | 1251.84 (934.85 to 1614.83) | -0.42 (-0.58 to 0.05) |
| Congo | 2152.98 (1948.79 to 2363) | 72.52 (65.77 to 79.84) | -0.38 (-0.41 to -0.34) |  | 1520.45 (1139.8 to 1978.73) | 64.55 (49.82 to 82.79) | -0.42 (-0.55 to -0.27) |  | 44257.22 (33214.27 to 58889.85) | 1502.43 (1145.45 to 1951.8) | -0.46 (-0.59 to -0.29) |
| Cook Islands | 15.22 (13.63 to 16.99) | 66.42 (59.82 to 73.49) | -0.18 (-0.23 to -0.13) |  | 8.06 (6.71 to 9.7) | 33.62 (27.94 to 40.65) | -0.48 (-0.58 to -0.34) |  | 198.93 (164.78 to 240.22) | 843.88 (688.99 to 1016.24) | -0.47 (-0.58 to -0.31) |
| Costa Rica | 873.47 (781.17 to 979.99) | 17.26 (15.45 to 19.39) | -0.31 (-0.35 to -0.26) |  | 450.4 (348.59 to 566.73) | 8.75 (6.74 to 11.04) | -0.52 (-0.64 to -0.38) |  | 10106.02 (7950.48 to 12771.78) | 195.81 (154.17 to 246.98) | -0.52 (-0.63 to -0.38) |
| Côte d'Ivoire | 11106.12 (10070.25 to 12248.71) | 89.76 (81.88 to 99.09) | -0.12 (-0.17 to -0.08) |  | 5933.26 (4459.07 to 7567.84) | 63.06 (49.78 to 77.35) | -0.19 (-0.36 to 0.01) |  | 183564.92 (134813.73 to 236467.58) | 1431.83 (1094.33 to 1802.12) | -0.22 (-0.4 to 0) |
| Croatia | 1536.02 (1419.94 to 1656.05) | 19.14 (17.73 to 20.68) | -0.57 (-0.59 to -0.55) |  | 1607.48 (1276.45 to 1994.86) | 17.68 (14.03 to 21.95) | -0.61 (-0.69 to -0.51) |  | 30931.33 (24689.92 to 38578.08) | 374.29 (298.58 to 467.1) | -0.66 (-0.73 to -0.57) |
| Cuba | 4259.75 (3908.23 to 4688.11) | 24.42 (22.31 to 27) | -0.34 (-0.38 to -0.3) |  | 3262.95 (2660.79 to 3927.21) | 16.89 (13.66 to 20.4) | -0.39 (-0.51 to -0.25) |  | 69760.18 (56218.22 to 84519.98) | 382.71 (308.22 to 463.52) | -0.43 (-0.54 to -0.31) |
| Cyprus | 262.97 (237.15 to 291.3) | 15.09 (13.64 to 16.71) | -0.48 (-0.51 to -0.44) |  | 215.78 (183.12 to 264.87) | 12.09 (10.25 to 14.84) | -0.65 (-0.71 to -0.57) |  | 3883.97 (3359.53 to 4627.99) | 208.21 (180.27 to 246.61) | -0.66 (-0.72 to -0.58) |
| Czechia | 2686.88 (2389.7 to 2990.22) | 14.84 (13.26 to 16.53) | -0.54 (-0.57 to -0.5) |  | 1930.9 (1572.28 to 2317.7) | 8.88 (7.27 to 10.69) | -0.71 (-0.76 to -0.64) |  | 37269.29 (30647.23 to 44742.58) | 188.88 (155.77 to 227.58) | -0.73 (-0.78 to -0.67) |
| Democratic People's Republic of Korea | 36472.13 (33597.46 to 39416.44) | 116.73 (108.12 to 125.4) | -0.02 (-0.06 to 0.02) |  | 33948.2 (28750.31 to 40321.79) | 111.46 (94.81 to 132.82) | -0.1 (-0.27 to 0.1) |  | 802521.92 (654433.52 to 965791.89) | 2484.66 (2039.65 to 2988.87) | -0.08 (-0.29 to 0.15) |
| Democratic Republic of the Congo | 26474.96 (23990.63 to 29161.06) | 66.02 (60 to 72.96) | -0.21 (-0.25 to -0.17) |  | 21650.34 (16124.27 to 28530.44) | 68.11 (50.25 to 89.27) | -0.22 (-0.39 to -0.01) |  | 613684.85 (464749.08 to 802150.77) | 1547.39 (1159.41 to 2022.98) | -0.24 (-0.42 to -0.02) |
| Denmark | 1220.37 (1096.78 to 1357.62) | 11.45 (10.28 to 12.85) | -0.49 (-0.53 to -0.46) |  | 1177.81 (1029.02 to 1315.93) | 9.47 (8.34 to 10.53) | -0.53 (-0.59 to -0.47) |  | 19551.64 (17536.88 to 21786.19) | 175.79 (158.71 to 195.08) | -0.58 (-0.62 to -0.52) |
| Djibouti | 386.55 (345.01 to 432.28) | 55.15 (49.53 to 61.5) | -0.38 (-0.41 to -0.34) |  | 287.39 (205.26 to 393.89) | 53.57 (40.03 to 70.72) | -0.34 (-0.52 to -0.09) |  | 8618.67 (5959.92 to 12104.95) | 1248.93 (910.01 to 1689.73) | -0.36 (-0.54 to -0.08) |
| Dominica | 27.43 (25 to 30.25) | 31.72 (28.94 to 35.05) | -0.21 (-0.25 to -0.17) |  | 23.46 (19.31 to 28.71) | 25.92 (21.27 to 31.72) | -0.31 (-0.46 to -0.12) |  | 506.15 (412.69 to 622.17) | 573.35 (467.06 to 705.25) | -0.32 (-0.46 to -0.14) |
| Dominican Republic | 4613.85 (4236.31 to 5079.33) | 47.85 (43.9 to 52.42) | 0.13 (0.08 to 0.19) |  | 3966.57 (2975.79 to 5303.21) | 43.45 (32.8 to 57.6) | 0.03 (-0.23 to 0.38) |  | 102304.68 (73919.51 to 137643.57) | 1057.57 (769.01 to 1422.75) | 0.03 (-0.25 to 0.41) |
| Ecuador | 3573 (3245.33 to 3982.34) | 22.89 (20.88 to 25.4) | -0.28 (-0.33 to -0.24) |  | 2041.2 (1615.37 to 2598.67) | 14.1 (11.21 to 17.8) | -0.51 (-0.61 to -0.36) |  | 53390.33 (42093.25 to 68390.97) | 339.32 (268.71 to 436.11) | -0.53 (-0.64 to -0.39) |
| Egypt | 27106.24 (24427.75 to 30213.97) | 39.62 (35.94 to 44.14) | -0.35 (-0.38 to -0.3) |  | 13653.66 (9489.02 to 19502.71) | 20.71 (14.46 to 30.07) | -0.56 (-0.69 to -0.39) |  | 525944.5 (380647.95 to 714427.6) | 641.8 (460.46 to 884.29) | -0.6 (-0.71 to -0.45) |
| El Salvador | 1323.95 (1196.55 to 1482.38) | 21.52 (19.39 to 24.19) | -0.36 (-0.4 to -0.32) |  | 882.55 (662.17 to 1182.04) | 14.3 (10.7 to 19.21) | -0.62 (-0.72 to -0.46) |  | 19786.54 (14844.45 to 26771.15) | 331.07 (248.36 to 449.02) | -0.65 (-0.75 to -0.5) |
| Equatorial Guinea | 302.57 (268.98 to 340.48) | 54.15 (48.46 to 60.87) | -0.54 (-0.57 to -0.51) |  | 194.64 (127.75 to 279.36) | 45.74 (30.82 to 62.9) | -0.58 (-0.72 to -0.36) |  | 5442.49 (3563.11 to 7941.66) | 1004.83 (667.81 to 1426.53) | -0.63 (-0.76 to -0.44) |
| Eritrea | 2227.32 (2035.38 to 2456.56) | 69.1 (63.34 to 76.35) | -0.4 (-0.43 to -0.37) |  | 1962.24 (1466.76 to 2540.18) | 81.41 (63.15 to 103.89) | -0.26 (-0.46 to 0) |  | 59776.97 (43799.5 to 79522.68) | 1913.15 (1445.44 to 2458.62) | -0.32 (-0.5 to -0.08) |
| Estonia | 316.14 (282.55 to 357.64) | 14.46 (12.88 to 16.55) | -0.64 (-0.67 to -0.61) |  | 200.68 (154.27 to 269.37) | 7.23 (5.52 to 9.67) | -0.76 (-0.82 to -0.68) |  | 4064.65 (3178.35 to 5346.51) | 172.13 (134.24 to 221.61) | -0.76 (-0.82 to -0.69) |
| Eswatini | 413.52 (378.04 to 455.75) | 63.92 (58.74 to 70.48) | -0.16 (-0.19 to -0.11) |  | 334.34 (238.87 to 454.86) | 63.97 (46.62 to 85.17) | -0.22 (-0.44 to 0.09) |  | 9437.41 (6601.24 to 13199.02) | 1483.06 (1063.2 to 2015.65) | -0.21 (-0.45 to 0.13) |
| Ethiopia | 20784.12 (17710.71 to 24352.66) | 43.86 (36.95 to 51.78) | -0.54 (-0.56 to -0.51) |  | 20931.84 (15749.02 to 26060.94) | 57.37 (42.04 to 71.96) | -0.51 (-0.66 to -0.33) |  | 534314.45 (410425.1 to 666008.3) | 1192.68 (908.21 to 1476.63) | -0.58 (-0.71 to -0.42) |
| Fiji | 460.63 (418.95 to 509.74) | 61.25 (56.15 to 67.49) | -0.24 (-0.28 to -0.2) |  | 325 (260.34 to 404.63) | 50.46 (41.18 to 61.96) | -0.35 (-0.5 to -0.12) |  | 9237.04 (7409.76 to 11515.18) | 1183.2 (963.57 to 1455.96) | -0.36 (-0.51 to -0.13) |
| Finland | 1643.64 (1461.23 to 1851.96) | 14.78 (13.09 to 16.59) | -0.23 (-0.29 to -0.17) |  | 1152.5 (997.75 to 1287.42) | 8.46 (7.4 to 9.45) | -0.46 (-0.55 to -0.4) |  | 19179.53 (17083.46 to 21456.01) | 166.65 (149.23 to 189.83) | -0.53 (-0.59 to -0.47) |
| France | 13953.88 (12619.76 to 15566.28) | 10.47 (9.45 to 11.67) | -0.4 (-0.45 to -0.36) |  | 12109.99 (10265.58 to 13605.85) | 7.35 (6.38 to 8.16) | -0.57 (-0.62 to -0.53) |  | 180448.97 (159830.06 to 198072.26) | 138.3 (123.93 to 151.68) | -0.61 (-0.65 to -0.58) |
| Gabon | 623.02 (560.47 to 693.69) | 55.56 (50.21 to 61.64) | -0.34 (-0.37 to -0.3) |  | 463.33 (345.27 to 578.25) | 48.02 (36.66 to 58.85) | -0.42 (-0.55 to -0.27) |  | 12650.94 (9285.89 to 16101.62) | 1117.4 (831.56 to 1399.14) | -0.44 (-0.58 to -0.28) |
| Gambia | 927.12 (842.96 to 1018.92) | 87.26 (79.1 to 96.8) | -0.03 (-0.07 to 0.02) |  | 617.68 (483.07 to 759.43) | 70.33 (56.2 to 84.76) | 0.05 (-0.22 to 0.39) |  | 16602.15 (12480.26 to 20991.49) | 1556.65 (1200.94 to 1929.33) | 0.02 (-0.26 to 0.4) |
| Georgia | 4138.08 (3857.73 to 4444.25) | 70.32 (65.75 to 75.46) | -0.38 (-0.42 to -0.35) |  | 4576.56 (3842.12 to 5367.43) | 72.49 (61.37 to 84.91) | -0.39 (-0.5 to -0.26) |  | 88181.68 (74389.39 to 103107.41) | 1527.05 (1287.45 to 1773.76) | -0.4 (-0.51 to -0.28) |
| Germany | 19015.19 (16930.53 to 21409.85) | 10.76 (9.55 to 12.19) | -0.44 (-0.48 to -0.4) |  | 15532.91 (13527.52 to 17348.53) | 7.22 (6.38 to 7.97) | -0.58 (-0.64 to -0.52) |  | 253505.82 (226660.71 to 277948.75) | 140.69 (128.06 to 153.6) | -0.62 (-0.67 to -0.57) |
| Ghana | 14715.37 (13360.45 to 16187.85) | 83.44 (75.96 to 91.52) | -0.01 (-0.06 to 0.04) |  | 9602.03 (7778.66 to 11707.92) | 66.44 (55.38 to 79.84) | -0.2 (-0.36 to 0.03) |  | 273766.75 (216263.55 to 337152.55) | 1517.39 (1224.13 to 1849.58) | -0.19 (-0.38 to 0.04) |
| Greece | 5731.92 (5236.28 to 6272.56) | 23.69 (21.61 to 26.04) | -0.45 (-0.48 to -0.42) |  | 6475.75 (5711.05 to 7077.72) | 22.89 (20.58 to 24.87) | -0.53 (-0.57 to -0.49) |  | 95320.21 (87016.28 to 102893.28) | 416.43 (384.96 to 448.92) | -0.52 (-0.56 to -0.48) |
| Greenland | 20.27 (18.65 to 22.34) | 31.59 (28.98 to 34.48) | -0.4 (-0.43 to -0.37) |  | 17.9 (14.59 to 21.52) | 29.32 (24.16 to 34.85) | -0.45 (-0.56 to -0.32) |  | 425.01 (346.65 to 513.51) | 616.66 (505.29 to 746.54) | -0.48 (-0.59 to -0.36) |
| Grenada | 39.49 (36.1 to 43.74) | 36.1 (33.05 to 39.85) | -0.32 (-0.36 to -0.28) |  | 34.8 (31.13 to 39.05) | 32.17 (28.87 to 35.96) | -0.49 (-0.57 to -0.39) |  | 862.01 (766.82 to 965.7) | 745.98 (667.09 to 833.77) | -0.51 (-0.59 to -0.42) |
| Guam | 102 (91.78 to 113.72) | 55.07 (49.68 to 61.18) | -0.08 (-0.13 to -0.02) |  | 47.29 (39.65 to 56.55) | 25.32 (21.24 to 30.1) | -0.45 (-0.55 to -0.31) |  | 1382.45 (1170.85 to 1629.64) | 735.97 (624.04 to 863.78) | -0.29 (-0.42 to -0.12) |
| Guatemala | 3633.98 (3317.15 to 4011.13) | 29.66 (27.1 to 32.52) | -0.41 (-0.44 to -0.37) |  | 2366.14 (1866.26 to 2961.95) | 22.64 (18.14 to 28.15) | -0.3 (-0.47 to -0.08) |  | 62923.95 (50312.09 to 79265.65) | 512.42 (409.52 to 645.19) | -0.39 (-0.53 to -0.21) |
| Guinea | 5390.44 (4940.52 to 5869.28) | 89.35 (81.72 to 97.55) | 0.08 (0.03 to 0.14) |  | 4024.71 (3201.68 to 4988.29) | 76.84 (61.97 to 94.39) | 0 (-0.22 to 0.29) |  | 114176.79 (88674.97 to 143242.5) | 1769.48 (1396.35 to 2205.37) | -0.01 (-0.25 to 0.28) |
| Guinea-Bissau | 1006.42 (922.82 to 1094.62) | 116.05 (106.52 to 125.83) | -0.14 (-0.17 to -0.1) |  | 633.12 (494.48 to 791.31) | 94.4 (75.35 to 115.59) | -0.15 (-0.36 to 0.16) |  | 20038.47 (15550.93 to 25468.47) | 2272.42 (1786.08 to 2826.85) | -0.19 (-0.41 to 0.11) |
| Guyana | 453 (422.97 to 486.11) | 68.31 (63.94 to 73.29) | -0.42 (-0.45 to -0.39) |  | 417.71 (317.36 to 527.65) | 68.37 (53.06 to 85.37) | -0.55 (-0.66 to -0.43) |  | 11360.85 (8659.5 to 14568.65) | 1675.46 (1288.4 to 2129.69) | -0.56 (-0.67 to -0.43) |
| Haiti | 6639.82 (6090.04 to 7236.61) | 84.17 (77.13 to 91.63) | -0.16 (-0.2 to -0.13) |  | 5229.29 (3397.2 to 7559.54) | 78.12 (50.79 to 112.37) | -0.33 (-0.49 to -0.12) |  | 153918.25 (100427.33 to 222366.83) | 1910.4 (1247.64 to 2752.09) | -0.36 (-0.53 to -0.14) |
| Honduras | 1470.67 (1307.84 to 1680.36) | 21.84 (19.58 to 24.62) | -0.21 (-0.26 to -0.16) |  | 2258.7 (1752.61 to 3003.24) | 39.68 (30.96 to 52.46) | 0.13 (-0.14 to 0.49) |  | 57337.79 (43838.95 to 77444.82) | 887.77 (686.57 to 1187.61) | -0.04 (-0.28 to 0.29) |
| Hungary | 3346.61 (3030.92 to 3683.66) | 20.11 (18.26 to 22.14) | -0.61 (-0.64 to -0.58) |  | 2527.98 (2086.5 to 3022.03) | 12.93 (10.74 to 15.52) | -0.73 (-0.78 to -0.68) |  | 52183.97 (43512.01 to 62547.87) | 298.72 (247.3 to 359.17) | -0.72 (-0.77 to -0.67) |
| Iceland | 49.25 (43.99 to 55.02) | 9.07 (8.09 to 10.2) | -0.46 (-0.5 to -0.42) |  | 34.42 (29.27 to 39.27) | 5.57 (4.79 to 6.33) | -0.63 (-0.68 to -0.57) |  | 562.81 (494.15 to 631.63) | 104.13 (91.59 to 116.87) | -0.66 (-0.7 to -0.61) |
| India | 626276.05 (523149.03 to 748002.91) | 52.98 (44.4 to 63.25) | -0.1 (-0.12 to -0.08) |  | 374136.33 (304424.81 to 449386.62) | 34.3 (27.85 to 41.26) | -0.41 (-0.52 to -0.29) |  | 9879695.62 (8226604.87 to 11827657.87) | 825.1 (679.35 to 985.45) | -0.38 (-0.49 to -0.26) |
| Indonesia | 274202.34 (231782.96 to 322944.05) | 119.37 (101.16 to 139.92) | -0.04 (-0.06 to -0.03) |  | 181933 (148175.52 to 216522.21) | 94.26 (77.03 to 113.57) | -0.08 (-0.23 to 0.09) |  | 4977770.42 (4114408.35 to 5860542.85) | 2166.22 (1788 to 2556.43) | -0.14 (-0.28 to 0.01) |
| Iran (Islamic Republic of) | 12797.69 (10809.44 to 15134.35) | 17.31 (14.57 to 20.59) | -0.28 (-0.3 to -0.25) |  | 6308.21 (5792.15 to 6704.8) | 9.29 (8.43 to 9.9) | -0.53 (-0.6 to -0.43) |  | 172625.94 (162359.4 to 183216.39) | 223.25 (209.58 to 236.27) | -0.54 (-0.6 to -0.47) |
| Iraq | 14714.17 (13481.41 to 16028.87) | 52.75 (48.22 to 57.91) | -0.23 (-0.27 to -0.18) |  | 9011.59 (7054.02 to 11185.5) | 40 (32.22 to 48.04) | -0.28 (-0.45 to -0.08) |  | 287202.21 (221960.67 to 364476.24) | 1022 (808.45 to 1271.13) | -0.3 (-0.47 to -0.09) |
| Ireland | 737.36 (653.6 to 825.15) | 10.71 (9.47 to 12.07) | -0.46 (-0.5 to -0.42) |  | 492 (425.07 to 552.44) | 6.35 (5.49 to 7.11) | -0.62 (-0.66 to -0.58) |  | 8672.48 (7714.35 to 9629.68) | 118.62 (105.76 to 132.16) | -0.65 (-0.68 to -0.61) |
| Israel | 1283.5 (1162.19 to 1428.34) | 11.21 (10.17 to 12.51) | -0.51 (-0.55 to -0.48) |  | 1172.27 (1028.72 to 1295.03) | 9.56 (8.48 to 10.5) | -0.6 (-0.65 to -0.55) |  | 20205.46 (18264.95 to 22050.29) | 177.72 (161.04 to 194.26) | -0.63 (-0.67 to -0.59) |
| Italy | 16553.91 (13794.17 to 19859.82) | 11.98 (10.19 to 14.17) | -0.42 (-0.45 to -0.38) |  | 18305.61 (15760.27 to 19940.72) | 10.7 (9.43 to 11.52) | -0.44 (-0.49 to -0.41) |  | 265918.7 (241474.54 to 282505.02) | 192.67 (178.89 to 202.81) | -0.53 (-0.56 to -0.51) |
| Jamaica | 1278.87 (1169.61 to 1397.14) | 42.59 (38.85 to 46.54) | 0.05 (-0.01 to 0.11) |  | 1087.84 (873.06 to 1333.31) | 36.15 (28.77 to 44.29) | -0.22 (-0.39 to 0) |  | 24880.92 (19692.32 to 31017.8) | 841.15 (665.22 to 1047.8) | -0.24 (-0.41 to -0.04) |
| Japan | 50869.78 (43196.88 to 59527.89) | 19.06 (16.15 to 22.3) | -0.4 (-0.44 to -0.35) |  | 38978.52 (31743.09 to 43153.78) | 9.41 (8.14 to 10.2) | -0.57 (-0.6 to -0.55) |  | 674766.46 (597867.6 to 732610.27) | 226.56 (208.63 to 244.35) | -0.52 (-0.54 to -0.5) |
| Jordan | 2161.5 (1916.5 to 2471.75) | 30.17 (26.87 to 34.03) | -0.4 (-0.43 to -0.35) |  | 819.63 (676.1 to 980.27) | 15.23 (12.57 to 18.11) | -0.58 (-0.67 to -0.48) |  | 22856.45 (19315.93 to 27054.49) | 325.61 (273.45 to 385.1) | -0.6 (-0.68 to -0.5) |
| Kazakhstan | 10741.72 (9936.56 to 11735.35) | 64.28 (59.49 to 70.02) | -0.28 (-0.32 to -0.23) |  | 8562.94 (7286.89 to 9836.97) | 56.03 (47.7 to 64.02) | -0.01 (-0.2 to 0.28) |  | 212589.18 (180324.21 to 246854.39) | 1211.18 (1032.23 to 1391.33) | -0.09 (-0.26 to 0.13) |
| Kenya | 15403.8 (13074.05 to 18069.94) | 61.17 (51.85 to 72.14) | -0.28 (-0.3 to -0.27) |  | 11768.45 (8658.09 to 14975.72) | 60.78 (44.33 to 77.01) | -0.12 (-0.23 to 0.02) |  | 325037.35 (243367.47 to 414416.42) | 1338.73 (992.68 to 1698.2) | -0.11 (-0.23 to 0.04) |
| Kiribati | 138.33 (128.5 to 148.63) | 161.75 (150.73 to 173.76) | -0.19 (-0.23 to -0.16) |  | 102.15 (80.46 to 126.27) | 152.17 (123.24 to 183.47) | -0.23 (-0.39 to -0.03) |  | 3474.23 (2731.91 to 4288.25) | 4152.38 (3304.69 to 5075.79) | -0.26 (-0.42 to -0.06) |
| Kuwait | 704.06 (626 to 802.97) | 21.42 (19.17 to 24.23) | -0.01 (-0.07 to 0.07) |  | 262.69 (206.86 to 319.31) | 10.77 (8.54 to 13.1) | -0.04 (-0.26 to 0.21) |  | 8431.74 (6893.17 to 10042.72) | 246.02 (201.3 to 294.02) | -0.04 (-0.23 to 0.18) |
| Kyrgyzstan | 2429.86 (2224.46 to 2671.49) | 47.99 (44.15 to 52.74) | -0.46 (-0.49 to -0.42) |  | 1761.61 (1520.39 to 2045.67) | 39.06 (33.75 to 44.9) | -0.38 (-0.48 to -0.27) |  | 50600.45 (43427.43 to 58891.79) | 994.01 (856.46 to 1151.39) | -0.4 (-0.49 to -0.29) |
| Lao People's Democratic Republic | 5367.52 (4934.75 to 5836.16) | 111.86 (102.87 to 121.25) | -0.3 (-0.33 to -0.27) |  | 3774.71 (3000.55 to 4754.64) | 93.87 (75.73 to 116.54) | -0.22 (-0.4 to 0.01) |  | 107760.35 (84639.12 to 137374.4) | 2233.14 (1782.52 to 2799.63) | -0.26 (-0.44 to -0.03) |
| Latvia | 800.99 (725.38 to 882.99) | 22.55 (20.47 to 24.86) | -0.56 (-0.59 to -0.53) |  | 671.69 (556.66 to 812.57) | 16.18 (13.36 to 19.82) | -0.63 (-0.7 to -0.55) |  | 13242.41 (11038.58 to 16156.88) | 365.83 (303.81 to 449.31) | -0.62 (-0.69 to -0.53) |
| Lebanon | 978.57 (866.3 to 1103.25) | 19.02 (16.89 to 21.45) | -0.44 (-0.47 to -0.4) |  | 256.69 (187.64 to 350.99) | 5.03 (3.67 to 6.9) | -0.69 (-0.78 to -0.55) |  | 6731.75 (5019.54 to 8958.52) | 128.39 (95.86 to 170.36) | -0.64 (-0.74 to -0.51) |
| Lesotho | 1198.34 (1106.48 to 1300.66) | 85.67 (79.15 to 92.96) | 0.11 (0.05 to 0.16) |  | 1116.97 (832.6 to 1460.62) | 98.65 (73.14 to 130.04) | 0.17 (-0.15 to 0.54) |  | 30169.85 (22371.58 to 39671.53) | 2243.48 (1681.64 to 2929.62) | 0.21 (-0.13 to 0.62) |
| Liberia | 1757.6 (1595.99 to 1939.37) | 73.57 (67.31 to 80.73) | -0.16 (-0.2 to -0.12) |  | 1076.19 (814.41 to 1431.73) | 58.47 (45.72 to 75.65) | -0.23 (-0.4 to -0.03) |  | 31220.82 (22887.43 to 41945.72) | 1295.2 (980.29 to 1732.75) | -0.28 (-0.45 to -0.06) |
| Libya | 1434.2 (1284.82 to 1592.33) | 25.29 (22.6 to 28.34) | -0.32 (-0.35 to -0.28) |  | 678.11 (497.39 to 935.72) | 13.26 (9.73 to 18.12) | -0.53 (-0.65 to -0.36) |  | 22582.6 (16898.43 to 30609.17) | 376.23 (284.93 to 504.38) | -0.51 (-0.63 to -0.34) |
| Lithuania | 979.25 (893.05 to 1090.28) | 19.73 (17.99 to 21.86) | -0.29 (-0.34 to -0.25) |  | 662.89 (547.15 to 793.45) | 11.35 (9.28 to 13.8) | -0.33 (-0.45 to -0.18) |  | 14139.82 (11616.38 to 17108.51) | 281.77 (230.71 to 341.97) | -0.38 (-0.49 to -0.24) |
| Luxembourg | 105.47 (95.95 to 114.93) | 10.67 (9.63 to 11.75) | -0.59 (-0.62 to -0.56) |  | 103.26 (85.9 to 119.44) | 9.44 (7.96 to 10.93) | -0.67 (-0.72 to -0.61) |  | 1687.33 (1450.42 to 1943.39) | 171.76 (147.86 to 196.7) | -0.71 (-0.75 to -0.66) |
| Madagascar | 15682.06 (14383.75 to 17197.69) | 114.68 (105.72 to 125.37) | -0.19 (-0.22 to -0.16) |  | 11051.41 (7972.66 to 14449.42) | 106.44 (78.48 to 137.94) | -0.14 (-0.33 to 0.13) |  | 351781.99 (256735.97 to 460146.04) | 2622.88 (1913.04 to 3397.18) | -0.18 (-0.38 to 0.06) |
| Malawi | 4504.24 (4065.36 to 5009.51) | 54.02 (49.01 to 59.89) | -0.26 (-0.3 to -0.22) |  | 3893.04 (2943.04 to 4893.18) | 57.8 (43.65 to 72.18) | -0.28 (-0.43 to -0.09) |  | 108839.18 (82260.34 to 140131.47) | 1321.44 (1006.35 to 1673.74) | -0.3 (-0.46 to -0.08) |
| Malaysia | 17232.43 (15682.17 to 19259.96) | 64.18 (58.39 to 71.09) | -0.4 (-0.44 to -0.37) |  | 10791.64 (8541.4 to 13477.06) | 44.77 (35.53 to 55.59) | -0.51 (-0.61 to -0.38) |  | 281125.34 (225294.36 to 347130.19) | 1016.45 (820.92 to 1256.68) | -0.53 (-0.62 to -0.41) |
| Maldives | 144.58 (129.8 to 160.66) | 42.05 (37.68 to 46.9) | -0.59 (-0.61 to -0.56) |  | 77.54 (64.22 to 92.71) | 28.54 (23.53 to 34.14) | -0.67 (-0.73 to -0.6) |  | 2106.05 (1739.35 to 2476.03) | 609.97 (511.56 to 720.53) | -0.71 (-0.77 to -0.65) |
| Mali | 7359.7 (6722.33 to 8047.52) | 78.72 (72.13 to 86.07) | -0.21 (-0.25 to -0.18) |  | 5525.21 (4392.06 to 6923.51) | 71.38 (56.62 to 87.96) | -0.18 (-0.35 to 0.02) |  | 162991.89 (126576.22 to 208830.05) | 1547.62 (1230.13 to 1942.57) | -0.23 (-0.39 to -0.03) |
| Malta | 113.92 (101.8 to 128.58) | 13.53 (12.15 to 15.17) | -0.46 (-0.5 to -0.43) |  | 86.37 (73.42 to 98.66) | 8.85 (7.59 to 10.12) | -0.66 (-0.71 to -0.61) |  | 1516.56 (1313.69 to 1729.17) | 178.55 (155.89 to 202.54) | -0.66 (-0.71 to -0.61) |
| Marshall Islands | 57.6 (53.17 to 62.88) | 143.98 (133.37 to 155.69) | -0.08 (-0.12 to -0.03) |  | 35.65 (25.98 to 47.57) | 116.44 (87.33 to 150.22) | -0.16 (-0.34 to 0.07) |  | 1160.61 (847.5 to 1541.74) | 2895.24 (2159.52 to 3771.75) | -0.14 (-0.33 to 0.11) |
| Mauritania | 1502.48 (1354.67 to 1675.29) | 67.93 (61.6 to 76.04) | -0.31 (-0.34 to -0.27) |  | 857.84 (664.12 to 1086.78) | 45.28 (36.05 to 56.04) | -0.42 (-0.55 to -0.26) |  | 22057.68 (16414.26 to 28827.52) | 986.06 (753.02 to 1263.98) | -0.47 (-0.6 to -0.31) |
| Mauritius | 666.22 (602.04 to 743.74) | 40.71 (37 to 44.96) | -0.52 (-0.55 to -0.49) |  | 392.78 (320.36 to 478.98) | 22.98 (18.75 to 27.88) | -0.63 (-0.71 to -0.55) |  | 10864.66 (8828.04 to 13211.98) | 622.94 (510.75 to 754.33) | -0.62 (-0.69 to -0.54) |
| Mexico | 23266.68 (19685.63 to 27359.09) | 19.61 (16.6 to 23.14) | -0.32 (-0.33 to -0.3) |  | 13214.45 (11366.61 to 15172.22) | 11.72 (10.08 to 13.47) | -0.42 (-0.5 to -0.34) |  | 332726.62 (286516.06 to 384253.03) | 276.88 (239.02 to 318.6) | -0.41 (-0.49 to -0.32) |
| Micronesia (Federated States of) | 97.6 (89.71 to 107.04) | 123.93 (114.65 to 135.12) | -0.22 (-0.25 to -0.18) |  | 75.01 (51.85 to 97.79) | 119.53 (87.82 to 152.23) | -0.18 (-0.41 to 0.1) |  | 2320.53 (1548.68 to 3090.87) | 2956.83 (2054.16 to 3856.43) | -0.2 (-0.45 to 0.1) |
| Monaco | 9.82 (8.75 to 11.1) | 11.49 (10.17 to 13.01) | -0.15 (-0.2 to -0.09) |  | 8.16 (6.38 to 10.09) | 7.68 (6.02 to 9.57) | -0.55 (-0.66 to -0.35) |  | 135.17 (106.88 to 167.16) | 159.33 (124.17 to 198.3) | -0.53 (-0.65 to -0.32) |
| Mongolia | 4087.68 (3826.93 to 4395.34) | 166.54 (156.48 to 179.06) | -0.38 (-0.41 to -0.35) |  | 3456.52 (2705.84 to 4454.71) | 178.48 (144.58 to 225) | 0.15 (-0.13 to 0.48) |  | 98265.4 (76695.63 to 127639.82) | 3840.99 (3025.94 to 4888.77) | 0.02 (-0.24 to 0.33) |
| Montenegro | 1169.96 (1084.05 to 1268.99) | 129.1 (119.69 to 139.59) | -0.14 (-0.18 to -0.1) |  | 1456.12 (1241.44 to 1661.56) | 158.23 (134.41 to 180.21) | -0.03 (-0.19 to 0.12) |  | 24098.15 (20572.41 to 27779.94) | 2505.46 (2141.17 to 2883.88) | -0.12 (-0.27 to 0.04) |
| Morocco | 10825.97 (9692.62 to 12111.7) | 33.67 (30.26 to 37.66) | -0.4 (-0.43 to -0.36) |  | 6700.2 (5089.89 to 8429.55) | 23.57 (18.37 to 29.11) | -0.51 (-0.64 to -0.35) |  | 181454.95 (138916.42 to 232630.34) | 553.99 (425.29 to 704.35) | -0.51 (-0.64 to -0.34) |
| Mozambique | 13629.61 (12381.54 to 15010.27) | 104.81 (95.61 to 115.32) | -0.04 (-0.09 to 0.01) |  | 9989.9 (7489.52 to 12823.14) | 95.77 (72.49 to 121.27) | -0.01 (-0.25 to 0.3) |  | 289415.95 (216515.88 to 371291.84) | 2329.38 (1757.48 to 2974.95) | 0.03 (-0.23 to 0.38) |
| Myanmar | 32277.08 (29549.9 to 35720.33) | 67.25 (62.01 to 74.19) | -0.32 (-0.35 to -0.28) |  | 46992.36 (39654.84 to 56054.71) | 109.62 (93.67 to 128.89) | -0.35 (-0.48 to -0.17) |  | 1237215.97 (1012480.98 to 1508356.34) | 2549.27 (2117.41 to 3076.52) | -0.41 (-0.55 to -0.22) |
| Namibia | 727.38 (661.08 to 804.44) | 49.47 (45.14 to 54.67) | -0.4 (-0.44 to -0.37) |  | 740.22 (576.03 to 950.78) | 57.47 (45.27 to 72.62) | -0.33 (-0.49 to -0.08) |  | 18254.75 (13947.38 to 23801.55) | 1252.56 (972.98 to 1610.15) | -0.35 (-0.52 to -0.1) |
| Nauru | 7.1 (6.48 to 7.76) | 117.87 (108.53 to 128.6) | -0.09 (-0.13 to -0.05) |  | 5.09 (3.84 to 6.53) | 122.68 (97.65 to 152.13) | -0.17 (-0.33 to 0.01) |  | 197.33 (148.08 to 256.38) | 3259.03 (2533.2 to 4093.53) | -0.15 (-0.31 to 0.04) |
| Nepal | 8097.96 (7200.54 to 9089.03) | 35.16 (31.33 to 39.33) | -0.27 (-0.3 to -0.23) |  | 7853.62 (5741.75 to 10213.96) | 38.29 (27.98 to 50.15) | -0.34 (-0.53 to -0.1) |  | 191375.78 (142270.5 to 247289.58) | 834.97 (618.26 to 1078.19) | -0.38 (-0.56 to -0.17) |
| Netherlands | 3720.43 (3351.86 to 4163.91) | 11.92 (10.69 to 13.37) | -0.38 (-0.42 to -0.34) |  | 3145.12 (2740.67 to 3505.47) | 8.55 (7.5 to 9.49) | -0.43 (-0.49 to -0.37) |  | 50948.07 (45888.66 to 55987.3) | 154.44 (140.12 to 168.97) | -0.49 (-0.54 to -0.44) |
| New Zealand | 626.42 (532.23 to 721.84) | 8.8 (7.55 to 10.25) | -0.4 (-0.46 to -0.33) |  | 619.56 (530.32 to 688.84) | 7.36 (6.35 to 8.17) | -0.47 (-0.53 to -0.41) |  | 10267.04 (9171.93 to 11288.64) | 135.62 (122.27 to 148.72) | -0.53 (-0.58 to -0.48) |
| Nicaragua | 1082.31 (967.68 to 1234.04) | 22.01 (19.75 to 24.68) | -0.38 (-0.42 to -0.33) |  | 780.88 (644.74 to 932.58) | 19.95 (16.67 to 23.4) | -0.4 (-0.51 to -0.25) |  | 18186.35 (14876.03 to 22093.15) | 405.29 (335.63 to 486) | -0.47 (-0.58 to -0.33) |
| Niger | 6943.98 (6286.44 to 7697.36) | 81.01 (73.79 to 89.42) | -0.14 (-0.18 to -0.09) |  | 5122.5 (3893.31 to 6700.95) | 73.3 (56.72 to 93.68) | -0.15 (-0.32 to 0.06) |  | 159490.76 (117183.95 to 213519.39) | 1633.33 (1254.42 to 2137.47) | -0.2 (-0.38 to 0.03) |
| Nigeria | 60091.94 (51125.35 to 70405.5) | 63.85 (54.09 to 75.21) | -0.29 (-0.3 to -0.28) |  | 39992.31 (31129.43 to 51487.06) | 52.17 (40.92 to 65.65) | -0.38 (-0.56 to -0.14) |  | 1116322.03 (863417.29 to 1458047.6) | 1124.44 (877.05 to 1450.18) | -0.38 (-0.56 to -0.14) |
| Niue | 1.59 (1.44 to 1.77) | 76.91 (69.96 to 85.33) | -0.15 (-0.19 to -0.11) |  | 1.39 (1.09 to 1.68) | 65.29 (51.5 to 79.35) | -0.3 (-0.45 to -0.11) |  | 33.35 (25.9 to 41.16) | 1587.63 (1232.06 to 1986.02) | -0.3 (-0.46 to -0.09) |
| North Macedonia | 1386.13 (1286.38 to 1508.98) | 48.36 (44.94 to 52.29) | -0.31 (-0.34 to -0.28) |  | 1648.25 (1315.37 to 1997.75) | 56.72 (45.93 to 67.99) | -0.26 (-0.41 to -0.09) |  | 34979.95 (27893.53 to 43072.89) | 1113.36 (892.18 to 1356.08) | -0.33 (-0.46 to -0.16) |
| Northern Mariana Islands | 39.66 (35.41 to 44.74) | 78.28 (71.02 to 86.56) | -0.21 (-0.25 to -0.16) |  | 26.05 (22.11 to 30.01) | 59 (50.84 to 67.5) | -0.39 (-0.5 to -0.27) |  | 742.98 (626.79 to 868.32) | 1371.53 (1168.4 to 1585.92) | -0.4 (-0.52 to -0.27) |
| Norway | 1050.64 (868.07 to 1269.17) | 11.54 (9.59 to 13.71) | -0.29 (-0.32 to -0.25) |  | 755.79 (662.48 to 833.74) | 7 (6.24 to 7.69) | -0.49 (-0.53 to -0.45) |  | 11894.67 (10914.55 to 12906.23) | 123.89 (113.92 to 134.44) | -0.56 (-0.58 to -0.53) |
| Oman | 669.07 (590.07 to 755.55) | 35.28 (31.44 to 39.56) | -0.39 (-0.43 to -0.35) |  | 322.77 (276.14 to 408.25) | 24.03 (20.39 to 30.49) | -0.57 (-0.68 to -0.4) |  | 10842.95 (9314.8 to 13403.79) | 504.15 (437.12 to 621.58) | -0.6 (-0.69 to -0.47) |
| Pakistan | 93345.26 (79367.73 to 109989.63) | 74.89 (64.06 to 88.21) | -0.16 (-0.19 to -0.14) |  | 56835.37 (47311.03 to 69007.59) | 54.76 (45.7 to 66.68) | -0.16 (-0.32 to 0.09) |  | 1698468.56 (1418193.18 to 2051124.35) | 1339.24 (1122.39 to 1618.64) | -0.12 (-0.3 to 0.13) |
| Palau | 16.89 (15.3 to 18.75) | 78.28 (71.71 to 85.67) | -0.1 (-0.15 to -0.05) |  | 12.08 (9.57 to 15.28) | 59.42 (48.18 to 73.6) | -0.28 (-0.47 to -0.03) |  | 377.46 (297.22 to 477.97) | 1635.73 (1302.27 to 2051.64) | -0.25 (-0.47 to 0.02) |
| Palestine | 726.82 (650.4 to 832.15) | 28.34 (25.79 to 31.28) | -0.39 (-0.43 to -0.35) |  | 468.08 (394.67 to 549.29) | 24.09 (20.29 to 28.03) | -0.56 (-0.66 to -0.42) |  | 12183.29 (10389.97 to 14188.8) | 487.33 (416.55 to 568.32) | -0.57 (-0.66 to -0.43) |
| Panama | 1015.94 (916.87 to 1139.12) | 24.26 (21.86 to 27.19) | -0.36 (-0.4 to -0.33) |  | 621.62 (475.02 to 796.89) | 14.86 (11.33 to 19.13) | -0.53 (-0.65 to -0.38) |  | 13976.21 (10846.11 to 18059.94) | 336.44 (261.38 to 433.59) | -0.53 (-0.65 to -0.39) |
| Papua New Guinea | 7396.97 (6760.44 to 8060.74) | 120.71 (110.85 to 130.99) | -0.09 (-0.13 to -0.05) |  | 3964.28 (2703.26 to 5586.98) | 95.05 (66.44 to 132.56) | -0.03 (-0.24 to 0.22) |  | 126735.3 (88219.91 to 173953.74) | 2284.91 (1599.87 to 3153.98) | -0.02 (-0.24 to 0.26) |
| Paraguay | 2180.98 (1967.84 to 2433.96) | 36.98 (33.61 to 41.03) | -0.34 (-0.38 to -0.29) |  | 1219.37 (925.8 to 1619.71) | 22.28 (16.87 to 29.53) | -0.51 (-0.64 to -0.33) |  | 31680.25 (24167.87 to 42289.18) | 546.45 (417.47 to 726.42) | -0.5 (-0.63 to -0.3) |
| Peru | 6216.53 (5609.51 to 6956.56) | 18.65 (16.8 to 20.86) | -0.44 (-0.47 to -0.4) |  | 3154.96 (2365.06 to 4246.84) | 9.67 (7.24 to 13.05) | -0.68 (-0.77 to -0.54) |  | 84228.04 (62567.56 to 113045.62) | 254.29 (189.48 to 339.26) | -0.67 (-0.77 to -0.54) |
| Philippines | 70640.22 (59564.14 to 82829.98) | 83.09 (70.76 to 97.24) | -0.11 (-0.12 to -0.1) |  | 44661.69 (35321.2 to 53185.16) | 59.75 (48.37 to 70.36) | 0.37 (0.04 to 0.69) |  | 1311982.09 (1043208.87 to 1555692.75) | 1522.32 (1214.29 to 1799.29) | 0.6 (0.1 to 0.98) |
| Poland | 11144.45 (9258.36 to 13261.62) | 17.82 (14.96 to 21.04) | -0.49 (-0.51 to -0.48) |  | 9891.14 (8283.04 to 11494.69) | 14.08 (11.79 to 16.41) | -0.58 (-0.64 to -0.51) |  | 208587.16 (174285.59 to 244451.2) | 326.17 (271.69 to 382.59) | -0.57 (-0.64 to -0.5) |
| Portugal | 3622.89 (3333.28 to 3940.4) | 15.71 (14.44 to 17.1) | -0.68 (-0.7 to -0.66) |  | 4235.92 (3757.06 to 4658.46) | 15.88 (14.18 to 17.36) | -0.71 (-0.74 to -0.68) |  | 69582.08 (63062.25 to 75686.73) | 308.11 (282.5 to 334.07) | -0.73 (-0.75 to -0.7) |
| Puerto Rico | 939.24 (847.48 to 1051.19) | 16.3 (14.58 to 18.47) | -0.38 (-0.42 to -0.33) |  | 632.11 (489.27 to 794.45) | 8.44 (6.47 to 10.63) | -0.47 (-0.59 to -0.31) |  | 12180.99 (9422.07 to 15584.37) | 194.9 (151.03 to 249.66) | -0.47 (-0.59 to -0.31) |
| Qatar | 384.74 (327.76 to 454.48) | 24.52 (21.87 to 27.6) | -0.34 (-0.38 to -0.29) |  | 77.21 (56.66 to 106.35) | 12.55 (9.77 to 16.26) | -0.49 (-0.64 to -0.28) |  | 3518.19 (2730.39 to 4574.18) | 243.59 (195.1 to 315.23) | -0.56 (-0.68 to -0.42) |
| Republic of Korea | 15112.58 (13645.16 to 17001.39) | 18.38 (16.59 to 20.63) | -0.77 (-0.79 to -0.75) |  | 10759.81 (9331.91 to 13438.63) | 12.68 (10.97 to 15.64) | -0.87 (-0.89 to -0.82) |  | 223518.73 (198961.92 to 283064.13) | 259.68 (232.46 to 330.72) | -0.87 (-0.89 to -0.82) |
| Republic of Moldova | 2186.35 (2024.28 to 2387.42) | 39.75 (36.83 to 43.19) | -0.53 (-0.56 to -0.5) |  | 1877.28 (1625.83 to 2161.43) | 32.13 (27.88 to 36.96) | -0.58 (-0.65 to -0.51) |  | 44805.36 (39095.66 to 51578.82) | 780.93 (682.14 to 900.31) | -0.56 (-0.63 to -0.49) |
| Romania | 12286.23 (11484.86 to 13220.3) | 35.66 (33.43 to 38.34) | -0.48 (-0.51 to -0.45) |  | 12916.28 (10776.37 to 15405.36) | 33.9 (28.21 to 40.71) | -0.4 (-0.53 to -0.23) |  | 256687.19 (213015.94 to 310221.11) | 737.51 (612.93 to 892.76) | -0.43 (-0.55 to -0.29) |
| Russian Federation | 70428.35 (59015.87 to 83398.98) | 32.6 (27.62 to 38.25) | -0.33 (-0.34 to -0.31) |  | 58086.17 (49730.65 to 66985.32) | 25.26 (21.59 to 29.14) | -0.35 (-0.43 to -0.25) |  | 1401941.45 (1207058.24 to 1621974.7) | 635.34 (546.64 to 735.6) | -0.29 (-0.39 to -0.16) |
| Rwanda | 3841.36 (3484.37 to 4244.99) | 61.03 (55.13 to 67.48) | -0.61 (-0.64 to -0.59) |  | 3670.06 (2552.06 to 5024.43) | 71.34 (47.81 to 98.06) | -0.52 (-0.65 to -0.38) |  | 96466.39 (67819.59 to 130976.74) | 1511.68 (1062.3 to 2055.85) | -0.59 (-0.7 to -0.45) |
| Saint Kitts and Nevis | 29.23 (27.08 to 31.73) | 44.8 (41.72 to 48.3) | -0.49 (-0.52 to -0.46) |  | 28.48 (23.3 to 33.67) | 46.56 (39.15 to 54.41) | -0.58 (-0.67 to -0.49) |  | 721.19 (562.37 to 878.66) | 1039.19 (824.02 to 1251.82) | -0.6 (-0.7 to -0.5) |
| Saint Lucia | 76.33 (70.5 to 83.37) | 36.4 (33.52 to 39.9) | -0.4 (-0.43 to -0.37) |  | 65.3 (55.03 to 76.91) | 31.02 (26.17 to 36.41) | -0.49 (-0.59 to -0.39) |  | 1528.17 (1282.58 to 1813.59) | 705.52 (592.63 to 834.98) | -0.5 (-0.59 to -0.39) |
| Saint Vincent and the Grenadines | 54.65 (50.47 to 59.74) | 41.94 (38.77 to 45.81) | 0.42 (0.34 to 0.5) |  | 45.81 (39.56 to 52.3) | 34.98 (30.22 to 39.77) | 0.14 (-0.02 to 0.34) |  | 1092.78 (937.88 to 1260.46) | 808.19 (693.54 to 928.93) | 0.11 (-0.06 to 0.31) |
| Samoa | 156.29 (143.64 to 170.93) | 100.94 (92.63 to 110.18) | -0.18 (-0.22 to -0.13) |  | 112.99 (90.56 to 138.19) | 82.73 (67.55 to 100.48) | -0.25 (-0.4 to -0.03) |  | 3067.23 (2359.45 to 3833.09) | 1985.5 (1556.69 to 2464.23) | -0.23 (-0.41 to 0.03) |
| San Marino | 7.11 (6.31 to 8.04) | 11.46 (10.19 to 12.87) | -0.27 (-0.31 to -0.22) |  | 6.48 (4.38 to 8.84) | 8.6 (5.77 to 11.95) | -0.37 (-0.6 to -0.09) |  | 99.13 (67.22 to 139.16) | 158.61 (107.1 to 225.91) | -0.37 (-0.6 to -0.07) |
| Sao Tome and Principe | 129 (118.22 to 141.95) | 108.28 (99.73 to 118.71) | 0 (-0.04 to 0.06) |  | 63.63 (49.84 to 79.17) | 66.77 (52.79 to 82.31) | -0.05 (-0.25 to 0.24) |  | 1794.06 (1402.57 to 2244.6) | 1517.47 (1199.42 to 1861.58) | -0.05 (-0.27 to 0.26) |
| Saudi Arabia | 9674.67 (8850.79 to 10619.41) | 42.81 (38.94 to 47.51) | -0.06 (-0.11 to -0.01) |  | 5292.01 (3981.37 to 6646.63) | 31.09 (24.01 to 37.42) | -0.46 (-0.61 to -0.22) |  | 192664.06 (149104.43 to 243126.23) | 764.7 (596.97 to 929.33) | -0.41 (-0.58 to -0.15) |
| Senegal | 5639.26 (5087.42 to 6239.93) | 69.93 (63.37 to 77.38) | -0.15 (-0.2 to -0.11) |  | 3891.88 (3073.97 to 4861.28) | 57.18 (45.83 to 70.12) | -0.14 (-0.32 to 0.09) |  | 105268.32 (81024.49 to 133039.86) | 1270 (997.1 to 1585.44) | -0.19 (-0.38 to 0.05) |
| Serbia | 4261.4 (3931.58 to 4637.75) | 30.21 (27.99 to 32.73) | -0.5 (-0.53 to -0.47) |  | 4458.66 (3625.01 to 5474.25) | 29.15 (23.7 to 35.55) | -0.53 (-0.63 to -0.41) |  | 85104.7 (68657.69 to 104441.4) | 560.87 (453.06 to 691.15) | -0.6 (-0.68 to -0.49) |
| Seychelles | 66.46 (59.69 to 73.76) | 58.68 (52.91 to 65.11) | -0.24 (-0.28 to -0.2) |  | 31.9 (27.15 to 38.1) | 29.86 (25.28 to 35.78) | -0.43 (-0.51 to -0.32) |  | 932.58 (806.01 to 1093.67) | 797.27 (690.57 to 934.68) | -0.43 (-0.51 to -0.33) |
| Sierra Leone | 3788.45 (3475.97 to 4147.33) | 93.57 (85.79 to 102.7) | -0.02 (-0.07 to 0.03) |  | 2384 (1754.06 to 3191.06) | 69.46 (52.47 to 91.67) | -0.08 (-0.29 to 0.2) |  | 74346.77 (53260.45 to 101412.38) | 1649.49 (1218.82 to 2202.78) | -0.1 (-0.33 to 0.21) |
| Singapore | 1233.31 (1105.83 to 1392.45) | 15.97 (14.26 to 18.04) | -0.62 (-0.65 to -0.58) |  | 550.35 (484.01 to 606.1) | 7.3 (6.38 to 8.06) | -0.75 (-0.77 to -0.72) |  | 14122.82 (12615.05 to 15527.95) | 179.61 (160.31 to 197.36) | -0.75 (-0.77 to -0.73) |
| Slovakia | 1514.23 (1385.71 to 1660.07) | 18.2 (16.67 to 19.96) | -0.51 (-0.54 to -0.48) |  | 1307.83 (1030.58 to 1631.36) | 14.32 (11.31 to 17.84) | -0.63 (-0.71 to -0.53) |  | 28345.53 (22336.65 to 35212.46) | 321.5 (253.95 to 400.07) | -0.64 (-0.72 to -0.54) |
| Slovenia | 511.68 (464.83 to 570.77) | 12.55 (11.41 to 13.89) | -0.54 (-0.58 to -0.51) |  | 460.05 (353.6 to 605.08) | 9.7 (7.47 to 12.65) | -0.58 (-0.71 to -0.42) |  | 8024.7 (6247.22 to 10264.38) | 194.23 (152.11 to 248.33) | -0.66 (-0.76 to -0.53) |
| Solomon Islands | 601.98 (551.56 to 655.1) | 156.2 (143.56 to 169.42) | -0.13 (-0.17 to -0.1) |  | 597.8 (480.66 to 742.34) | 214.62 (177.64 to 255.29) | 0.01 (-0.22 to 0.3) |  | 18904.46 (14987.18 to 23922.44) | 5250.9 (4266.16 to 6444.23) | -0.03 (-0.27 to 0.26) |
| Somalia | 7195.66 (6564.02 to 7933.26) | 87.66 (80.23 to 96.58) | -0.32 (-0.35 to -0.3) |  | 5538.14 (3863.19 to 7702.17) | 90.37 (63.75 to 123.52) | -0.25 (-0.45 to 0.04) |  | 172727.34 (119967.28 to 237835) | 2159.97 (1518.26 to 2972.36) | -0.28 (-0.48 to 0.02) |
| South Africa | 19041.67 (15975.81 to 22672.15) | 42.05 (35.34 to 49.85) | -0.24 (-0.28 to -0.21) |  | 12440.93 (11491.64 to 13366.93) | 29.77 (27.57 to 31.93) | -0.26 (-0.34 to -0.16) |  | 328097.55 (301983.24 to 354517.02) | 694.2 (640.79 to 747.74) | -0.35 (-0.41 to -0.27) |
| South Sudan | 2857.14 (2558.27 to 3159.1) | 63.93 (57.7 to 70.94) | -0.31 (-0.35 to -0.27) |  | 1925.07 (1318.55 to 2629.1) | 56.32 (39.86 to 75.5) | -0.35 (-0.53 to -0.11) |  | 54365.35 (36959.25 to 75970.41) | 1261.29 (882.01 to 1717.03) | -0.38 (-0.56 to -0.12) |
| Spain | 11186.14 (10126.58 to 12385.19) | 11.71 (10.58 to 13.02) | -0.52 (-0.55 to -0.49) |  | 11171.24 (9596.89 to 12625.65) | 9.8 (8.56 to 10.93) | -0.6 (-0.64 to -0.55) |  | 167588.67 (149849.67 to 183437.75) | 180.12 (163.04 to 195.25) | -0.65 (-0.68 to -0.62) |
| Sri Lanka | 7253.99 (6537.55 to 8062.91) | 30.88 (27.92 to 34.47) | -0.35 (-0.39 to -0.31) |  | 4019.71 (2918.81 to 5365.63) | 17.19 (12.7 to 22.74) | -0.51 (-0.65 to -0.32) |  | 103582.54 (77761.96 to 137317.45) | 409.26 (310.87 to 539.3) | -0.48 (-0.62 to -0.3) |
| Sudan | 10579.79 (9644.93 to 11598.9) | 45.75 (41.59 to 50.22) | -0.44 (-0.47 to -0.41) |  | 5659.65 (3818.04 to 7962.74) | 30.43 (20.87 to 42.2) | -0.62 (-0.7 to -0.49) |  | 185672.63 (126437.07 to 260180.72) | 769.89 (528.15 to 1070.27) | -0.61 (-0.7 to -0.48) |
| Suriname | 353.18 (325.31 to 385.96) | 58.1 (53.57 to 63.57) | -0.14 (-0.19 to -0.1) |  | 294.21 (244.2 to 353.62) | 49.88 (41.47 to 59.94) | -0.19 (-0.34 to -0.03) |  | 7475.96 (6168.57 to 9092.62) | 1209.13 (1004.85 to 1463.9) | -0.2 (-0.35 to -0.03) |
| Sweden | 2802.44 (2284.55 to 3417.31) | 14.6 (12.16 to 17.43) | -0.26 (-0.3 to -0.22) |  | 1919.72 (1698.12 to 2138.75) | 7.88 (7.08 to 8.71) | -0.41 (-0.46 to -0.35) |  | 29965.14 (27250.03 to 32821.38) | 146.82 (134.2 to 159.21) | -0.46 (-0.5 to -0.42) |
| Switzerland | 1368.19 (1213.2 to 1537.7) | 8.11 (7.12 to 9.12) | -0.54 (-0.58 to -0.5) |  | 1035.3 (871.16 to 1218.59) | 5.01 (4.24 to 5.82) | -0.64 (-0.7 to -0.57) |  | 15386.75 (13373.49 to 17486.7) | 87.71 (77.31 to 99.04) | -0.67 (-0.72 to -0.62) |
| Syrian Arab Republic | 5744.73 (5230.91 to 6338.49) | 47.39 (43.27 to 51.96) | -0.34 (-0.38 to -0.3) |  | 3895.18 (2952.36 to 5174.28) | 37.88 (29.09 to 49.37) | -0.42 (-0.58 to -0.2) |  | 106469.45 (80729.35 to 141598.01) | 838.99 (646.44 to 1101.7) | -0.48 (-0.63 to -0.29) |
| Taiwan (Province of China) | 12123.96 (10894 to 13472.52) | 33.04 (29.72 to 36.63) | -0.61 (-0.63 to -0.58) |  | 7028.15 (5614.04 to 8892.8) | 17.85 (14.22 to 22.65) | -0.75 (-0.8 to -0.68) |  | 161775.7 (128932.75 to 203518.3) | 430.12 (344.15 to 542.03) | -0.72 (-0.78 to -0.65) |
| Tajikistan | 4022.51 (3638.89 to 4494.05) | 79.39 (72.12 to 88.15) | -0.12 (-0.17 to -0.06) |  | 4727.19 (3774.12 to 5801.92) | 131.07 (106.07 to 157.62) | 0.25 (0.03 to 0.52) |  | 117387.41 (93577.17 to 145141.45) | 2525.46 (2031.88 to 3078.15) | 0.09 (-0.11 to 0.33) |
| Thailand | 39880.21 (36214.01 to 43864.54) | 41.4 (37.79 to 45.51) | -0.47 (-0.5 to -0.44) |  | 23738.6 (17797.6 to 31055.43) | 24.15 (18.1 to 31.36) | -0.6 (-0.7 to -0.46) |  | 621631.23 (467736.79 to 811194.8) | 641.37 (487.21 to 826.6) | -0.53 (-0.65 to -0.37) |
| Timor-Leste | 908.91 (834.2 to 997.27) | 109.54 (100.55 to 119.83) | -0.08 (-0.12 to -0.03) |  | 662.99 (493.3 to 884.25) | 88.19 (66.42 to 116.5) | 0.1 (-0.16 to 0.41) |  | 17393.54 (12949.48 to 23482.65) | 2031.08 (1513.02 to 2732.24) | 0.09 (-0.2 to 0.41) |
| Togo | 3682.64 (3354.49 to 4049.98) | 90.66 (82.98 to 100.02) | -0.06 (-0.11 to -0.02) |  | 2193.37 (1697.15 to 2793.1) | 67.49 (54.48 to 83.89) | -0.11 (-0.29 to 0.13) |  | 66136.81 (49755.5 to 84906.47) | 1553.91 (1210.41 to 1969.72) | -0.13 (-0.32 to 0.12) |
| Tokelau | 1 (0.91 to 1.11) | 77.14 (70.5 to 85.1) | -0.21 (-0.25 to -0.17) |  | 0.84 (0.66 to 1.05) | 66.67 (53.11 to 82.56) | -0.36 (-0.5 to -0.16) |  | 21.45 (16.36 to 27.26) | 1609.68 (1237.07 to 2042.77) | -0.34 (-0.51 to -0.11) |
| Tonga | 45.72 (41.62 to 50.6) | 55.64 (50.61 to 61.56) | -0.21 (-0.25 to -0.17) |  | 31.24 (25.66 to 38.04) | 40.53 (33.3 to 49.18) | -0.2 (-0.38 to 0.05) |  | 735.26 (602.33 to 904.22) | 914.72 (751.3 to 1118.52) | -0.19 (-0.37 to 0.06) |
| Trinidad and Tobago | 478.53 (436.28 to 530.5) | 27.5 (25.07 to 30.42) | -0.39 (-0.42 to -0.35) |  | 384.61 (287.1 to 504.54) | 20.89 (15.58 to 27.48) | -0.54 (-0.66 to -0.39) |  | 9143.27 (6783.53 to 12168.98) | 492.53 (366.81 to 652.06) | -0.54 (-0.67 to -0.38) |
| Tunisia | 2884.13 (2533.48 to 3290.38) | 23.94 (21.1 to 27.31) | -0.38 (-0.41 to -0.34) |  | 1691.88 (1254.34 to 2246.51) | 14.48 (10.81 to 19.06) | -0.59 (-0.72 to -0.41) |  | 40606.99 (29900.97 to 53482.63) | 322.51 (238.92 to 422.48) | -0.56 (-0.69 to -0.39) |
| Turkey | 30027.02 (26922.96 to 33453.4) | 35.06 (31.41 to 39.08) | -0.12 (-0.18 to -0.06) |  | 15611.02 (12430.46 to 19184.29) | 18.7 (14.95 to 22.94) | -0.31 (-0.49 to -0.1) |  | 350826.06 (284141.45 to 427495.9) | 399.02 (323.73 to 485.57) | -0.37 (-0.54 to -0.2) |
| Turkmenistan | 3341.95 (3062.78 to 3670.81) | 79.22 (72.72 to 87.15) | -0.13 (-0.18 to -0.09) |  | 2308.23 (1822.89 to 2938.53) | 59.75 (47.59 to 76.1) | -0.03 (-0.24 to 0.25) |  | 68527.11 (54173.42 to 86418.3) | 1570.21 (1252.13 to 1978.34) | -0.01 (-0.22 to 0.27) |
| Tuvalu | 9.5 (8.72 to 10.32) | 92.82 (85.37 to 100.85) | -0.18 (-0.21 to -0.14) |  | 8.94 (7.03 to 11.37) | 94.05 (74.84 to 118.58) | -0.32 (-0.49 to -0.08) |  | 245.8 (189.94 to 314.07) | 2322.29 (1813.41 to 2961.02) | -0.32 (-0.5 to -0.07) |
| Uganda | 8818.79 (7963.17 to 9871.83) | 54.58 (49.27 to 60.62) | -0.38 (-0.42 to -0.35) |  | 7844.22 (5826.14 to 10044.79) | 61.52 (44.19 to 78.55) | -0.22 (-0.39 to -0.02) |  | 219354.97 (166180.18 to 283594.79) | 1365.36 (1028.7 to 1744.04) | -0.24 (-0.42 to -0.01) |
| Ukraine | 24442.43 (20541.14 to 29097.08) | 37.23 (31.36 to 43.78) | -0.16 (-0.2 to -0.11) |  | 17533.02 (14900.56 to 20934.49) | 24.1 (20.33 to 28.9) | -0.07 (-0.26 to 0.27) |  | 481070.07 (406459.52 to 574787.36) | 709.86 (597.79 to 847.27) | 0.04 (-0.16 to 0.36) |
| United Arab Emirates | 2561.09 (2256.7 to 2883.32) | 42.29 (38.11 to 47.01) | -0.42 (-0.45 to -0.39) |  | 811.71 (533.82 to 1256.96) | 17.86 (12.48 to 27.19) | -0.68 (-0.78 to -0.54) |  | 35021.1 (23806.8 to 53068.67) | 449.82 (317.92 to 664.79) | -0.64 (-0.75 to -0.49) |
| United Kingdom | 13603.01 (11522.87 to 16077.73) | 11.42 (9.76 to 13.35) | -0.33 (-0.36 to -0.31) |  | 12393.85 (11150.47 to 13236.33) | 8.95 (8.13 to 9.55) | -0.37 (-0.4 to -0.33) |  | 199515.46 (184841.11 to 212120.65) | 166.15 (155.39 to 177.38) | -0.44 (-0.47 to -0.42) |
| United Republic of Tanzania | 15124.79 (13587.73 to 16915.38) | 54.61 (49.38 to 61.09) | -0.27 (-0.31 to -0.23) |  | 13112.03 (9964.63 to 17065.68) | 57.85 (43.92 to 74.85) | -0.27 (-0.43 to -0.08) |  | 354692.65 (268157.56 to 470397.38) | 1299.3 (989.6 to 1681.05) | -0.31 (-0.47 to -0.11) |
| United States of America | 71730.51 (58954.86 to 86204) | 13.76 (11.43 to 16.47) | -0.16 (-0.18 to -0.15) |  | 59730.58 (54339.32 to 64893.95) | 10.34 (9.5 to 11.23) | -0.12 (-0.16 to -0.07) |  | 1194222.12 (1119886.79 to 1285472.04) | 231.74 (218.48 to 249.45) | -0.15 (-0.19 to -0.11) |
| United States Virgin Islands | 44.61 (40.46 to 49.41) | 28.11 (25.62 to 31.01) | -0.11 (-0.16 to -0.06) |  | 39.95 (34.21 to 46.47) | 21.9 (18.72 to 25.36) | -0.2 (-0.35 to 0) |  | 894.35 (756.73 to 1046.75) | 498.74 (419.34 to 587.48) | -0.21 (-0.37 to 0) |
| Uruguay | 999.4 (907.67 to 1103.27) | 19.9 (18.09 to 22.07) | -0.28 (-0.32 to -0.23) |  | 798.44 (710.18 to 878.46) | 13.98 (12.62 to 15.31) | -0.41 (-0.48 to -0.33) |  | 15673.26 (14220.24 to 17076.89) | 313.2 (284.72 to 340.65) | -0.47 (-0.52 to -0.39) |
| Uzbekistan | 16883.89 (15691.94 to 18334.75) | 87.81 (81.92 to 94.52) | -0.01 (-0.06 to 0.05) |  | 13269.18 (11219.32 to 15707.8) | 89.53 (77.39 to 102.48) | 0.37 (0.15 to 1.13) |  | 372417.66 (315416.63 to 442897.26) | 1791.24 (1545.96 to 2088.04) | 0.2 (0 to 0.64) |
| Vanuatu | 274.95 (254.2 to 298.54) | 142.66 (131.86 to 154.76) | -0.14 (-0.18 to -0.1) |  | 196.45 (147.13 to 265.45) | 120.52 (90.46 to 159) | -0.08 (-0.29 to 0.22) |  | 5988.63 (4456.27 to 8074.18) | 3074.47 (2319.21 to 4089.14) | -0.05 (-0.28 to 0.31) |
| Venezuela (Bolivarian Republic of) | 9302.71 (8469.17 to 10298.05) | 32.09 (29.19 to 35.47) | -0.32 (-0.35 to -0.28) |  | 7471.49 (5703.84 to 9678.78) | 26.31 (20.2 to 33.87) | -0.3 (-0.47 to -0.07) |  | 180592.56 (136616.53 to 235793.95) | 608.19 (461.51 to 793.74) | -0.31 (-0.48 to -0.09) |
| Viet Nam | 84203.34 (77602.01 to 91468.89) | 87.67 (81.13 to 95.21) | -0.11 (-0.15 to -0.07) |  | 69121.2 (55195.11 to 85538.8) | 79.9 (63.75 to 97.95) | -0.23 (-0.42 to 0.01) |  | 1758557.07 (1408285.41 to 2184317.23) | 1817.21 (1460.53 to 2251.79) | -0.23 (-0.43 to 0.03) |
| Yemen | 7447.38 (6769.99 to 8164.89) | 44.75 (40.7 to 48.82) | -0.46 (-0.49 to -0.43) |  | 4607.39 (3394.82 to 6206.16) | 36.03 (26.84 to 46.79) | -0.56 (-0.67 to -0.41) |  | 146683.72 (108214.13 to 195409.32) | 884.3 (663.17 to 1178.86) | -0.54 (-0.66 to -0.37) |
| Zambia | 4390.24 (3979.68 to 4845.46) | 55.69 (50.66 to 61.16) | -0.29 (-0.32 to -0.25) |  | 5703.47 (4463.79 to 7109.37) | 95.5 (75.07 to 118.09) | 0.02 (-0.23 to 0.31) |  | 158343.57 (122373.89 to 199844.61) | 2128.82 (1661.38 to 2652.4) | 0 (-0.26 to 0.29) |
| Zimbabwe | 3126.34 (2826.48 to 3479.41) | 38.67 (35.19 to 42.73) | 0.24 (0.18 to 0.3) |  | 2142.88 (1644.74 to 2787) | 32.83 (25.7 to 42.93) | -0.01 (-0.24 to 0.28) |  | 63016.65 (48214.9 to 81480.14) | 798.46 (612.98 to 1028.23) | 0.05 (-0.19 to 0.36) |
