# Supplementary material for: Intracerebral Hemorrhage: The Global Differential Burden and Secular Trends From 1990 to 2019 and Its Prediction up to 2030
Source: Int J Public Health. 2025 May 21;70:1607013. doi: 10.3389/ijph.2025.1607013 (PMC12133604; doi:10.3389/ijph.2025.1607013)
Supplement: Supplementary file 9 [file Table3.docx]

Supplementary Table S3: Death number, age standardized mortality rate in 1990 and 2019 the percentage change of death number for intracerebral hemorrhage between 1990 and 2019 with EAPC.

| location | Death number  in 1990 | Death number  in 2019 | ASMR  in 2019 | EAPC  1990 to 2019 | Change of death number between 1990 and 2019 |
| --- | --- | --- | --- | --- | --- |
| Global  Both sexes | 2099761 (1932531 to 2328411) | 2886196 (2644484 to 3099351) | 36.04 (32.98 to 38.67) | -1.58% (-1.81 to -1.36) | 37.5% |
| Male | 1078052 (983324 to 1207351) | 1571625 (1411917 to 1719446) | 42.89 (38.6 to 46.87) | -1.4% (-1.63 to -1.16) | 45.8% |
| Female | 1021708 (909947 to 1142312) | 1314571 (1169674 to 1451009) | 30.06 (26.75 to 33.18) | -1.83% (-2.05 to -1.62) | 28.7% |
| Region  Australasia | 3213 (2983 to 3402) | 3726 (3220 to 4106) | 6.84 (5.99 to 7.52) | -2.64% (-2.74 to -2.55) | 16% |
| Caribbean | 11423 (10473 to 12474) | 16386 (13840 to 19150) | 31.73 (26.81 to 37.07) | -1.13% (-1.26 to -1) | 43.4% |
| Central Asia | 32407 (27864 to 34446) | 46335 (42039 to 51002) | 73.28 (66.72 to 80.05) | -0.35% (-0.88 to 0.19) | 43% |
| Central Europe | 69506 (66361 to 72171) | 51023 (44453 to 57689) | 23.34 (20.32 to 26.39) | -3.3% (-3.59 to -3) | -26.6% |
| Central Latin America | 21093 (20085 to 21948) | 33436 (28779 to 39029) | 14.48 (12.46 to 16.88) | -2.59% (-2.76 to -2.43) | 58.5% |
| Central Sub-Saharan Africa | 19320 (15882 to 23331) | 32118 (25030 to 41047) | 67.38 (51.77 to 85.65) | -1.11% (-1.18 to -1.03) | 66.2% |
| East Asia | 804882 (710906 to 971214) | 1110098 (962980 to 1277569) | 59.81 (52.14 to 68.34) | -2.15% (-2.58 to -1.71) | 37.9% |
| Eastern Europe | 94574 (88297 to 98295) | 82636 (73062 to 92611) | 24.51 (21.62 to 27.53) | -2.08% (-2.65 to -1.52) | -12.6% |
| Eastern Sub-Saharan Africa | 70430 (59559 to 81209) | 101232 (83168 to 120864) | 68.32 (55.63 to 81.68) | -1.4% (-1.46 to -1.35) | 43.7% |
| Andean Latin America | 6824 (5917 to 7687) | 7393 (6017 to 9142) | 13.32 (10.82 to 16.45) | -3.25% (-3.52 to -2.99) | 8.3% |
| High-income Asia Pacific | 59123 (55437 to 62132) | 50350 (41616 to 56156) | 9.85 (8.49 to 10.84) | -4.07% (-4.25 to -3.9) | -14.8% |
| High-income North America | 41743 (39104 to 43384) | 64359 (58276 to 69748) | 9.9 (9.08 to 10.72) | -0.84% (-1.02 to -0.66) | 54.2% |
| North Africa and Middle East | 75021 (66397 to 86132) | 88542 (77871 to 101100) | 21.59 (18.98 to 24.33) | -2.63% (-2.73 to -2.53) | 18% |
| Oceania | 2691 (2095 to 3450) | 5853 (4419 to 7604) | 91.45 (69.85 to 117.95) | -0.32% (-0.36 to -0.29) | 117.5% |
| South Asia | 315176 (266139 to 370472) | 521307 (442787 to 608642) | 39.34 (33.15 to 45.8) | -1.8% (-1.95 to -1.65) | 65.4% |
| Southeast Asia | 218747 (194980 to 243855) | 395691 (351745 to 444295) | 69.44 (61.5 to 77.87) | -0.85% (-0.99 to -0.71) | 80.9% |
| Southern Latin America | 18791 (17476 to 20050) | 14674 (13521 to 15950) | 17.52 (16.14 to 18.99) | -3.28% (-3.48 to -3.09) | -21.9% |
| Southern Sub-Saharan Africa | 11579 (10333 to 12816) | 17480 (15998 to 19071) | 33.27 (30.48 to 36.11) | -0.91% (-1.43 to -0.38) | 51% |
| Tropical Latin America | 45532 (43378 to 47686) | 45045 (41807 to 47658) | 18.73 (17.33 to 19.82) | -3.64% (-3.76 to -3.53) | -1.1% |
| Western Europe | 112835 (106525 to 117780) | 95748 (84497 to 103419) | 9.13 (8.2 to 9.78) | -2.85% (-2.97 to -2.74) | -15.1% |
| Western Sub-Saharan Africa | 64849 (53635 to 77301) | 102766 (86656 to 120418) | 60.72 (51.73 to 70.42) | -1.04% (-1.13 to -0.96) | 58.5% |
