# Supplementary material for: Intracerebral Hemorrhage: The Global Differential Burden and Secular Trends From 1990 to 2019 and Its Prediction up to 2030
Source: Int J Public Health. 2025 May 21;70:1607013. doi: 10.3389/ijph.2025.1607013 (PMC12133604; doi:10.3389/ijph.2025.1607013)
Supplement: Supplementary file 10 [file Table4.docx]

Supplementary Table 4: DALY number, age-standardized DALY rate in 1990 and 2019 the percentage change of DALY number for intracerebral hemorrhage between 1990 and 2019 with EAPC.

| location | DALY number  in 1990 | DALY number  in 2019 | ASDR  in 2019 | EAPC  1990 to 2019 | Change of DALY number between 1990 and 2019 |
| --- | --- | --- | --- | --- | --- |
| Global  Both sexes | 54726597 (50748682 to 59624631) | 68572498 (63272310 to 73681973) | 832.77 (769.21 to 894.68) | -1.64% (-1.82 to -1.46) | 25.3% |
| Male | 29621163 (26989496 to 32941132) | 39336303 (35415118 to 42947067) | 1001.11 (902.02 to 1092.01) | -1.41% (-1.61 to -1.22) | 32.8% |
| Female | 25105435 (22771411 to 27503101) | 29236195 (26330706 to 32021340) | 676.79 (610.14 to 741.11) | -1.95% (-2.13 to -1.77) | 16.5% |
| Region  Australasia | 64661 (60976 to 67800) | 59529 (53673 to 64797) | 122.72 (111.89 to 133.13) | -3.01% (-3.14 to -2.89) | -7.9% |
| Caribbean | 317113 (289938 to 347631) | 418562 (349077 to 496819) | 817.26 (682.22 to 971.05) | -1.14% (-1.31 to -0.97) | 32% |
| Central Asia | 809098 (730129 to 852414) | 1180303 (1063959 to 1305921) | 1564.2 (1418.02 to 1725.07) | -0.64% (-1.12 to -0.15) | 45.9% |
| Central Europe | 1618757 (1560326 to 1676169) | 996536 (868194 to 1125032) | 494.38 (432.51 to 558.61) | -3.56% (-3.88 to -3.24) | -38.4% |
| Central Latin America | 600542 (576751 to 622774) | 813578 (699129 to 945837) | 337.77 (290.54 to 392.74) | -2.59% (-2.76 to -2.42) | 35.5% |
| Central Sub-Saharan Africa | 594644 (491172 to 706231) | 931118 (731595 to 1171990) | 1551.36 (1216.01 to 1970.98) | -1.25% (-1.32 to -1.18) | 56.6% |
| East Asia | 19638775 (17342730 to 23057974) | 23174853 (19912136 to 26698566) | 1147.92 (987.87 to 1319.93) | -2.25% (-2.63 to -1.87) | 18% |
| Eastern Europe | 2337528 (2188719 to 2439510) | 2046121 (1818373 to 2291724) | 639.18 (567.16 to 716.52) | -1.81% (-2.43 to -1.18) | -12.5% |
| Eastern Sub-Saharan Africa | 2091020 (1780677 to 2393174) | 2836113 (2356451 to 3399186) | 1544.72 (1274.47 to 1833.27) | -1.65% (-1.72 to -1.58) | 35.6% |
| Andean Latin America | 206733 (176104 to 233257) | 193266 (156232 to 238397) | 331.56 (268.41 to 408.96) | -3.36% (-3.62 to -3.11) | -6.5% |
| High-income Asia Pacific | 1408327 (1339516 to 1470408) | 914146 (819289 to 1000461) | 230.08 (212.64 to 251.95) | -3.99% (-4.14 to -3.84) | -35.1% |
| High-income North America | 902188 (863301 to 937191) | 1274981 (1195203 to 1369691) | 220.15 (207.33 to 236.45) | -0.9% (-1.06 to -0.75) | 41.3% |
| North Africa and Middle East | 2614545 (2329415 to 3005133) | 2702244 (2343718 to 3120307) | 548.43 (479.52 to 623.09) | -2.6% (-2.68 to -2.53) | 3.4% |
| Oceania | 83950 (66078 to 105925) | 184217 (141778 to 237200) | 2250.67 (1745.22 to 2886.12) | -0.26% (-0.3 to -0.22) | 119.4% |
| South Asia | 8826818 (7512396 to 10205648) | 13740566 (11774657 to 15890550) | 931.36 (798.12 to 1075.7) | -1.58% (-1.68 to -1.48) | 55.7% |
| Southeast Asia | 6326361 (5733762 to 6920543) | 10675277 (9465511 to 11929104) | 1661.64 (1473.86 to 1853.11) | -0.86% (-0.98 to -0.73) | 68.7% |
| Southern Latin America | 484605 (459134 to 509707) | 331376 (307858 to 355587) | 411.02 (381.82 to 440.56) | -3.55% (-3.75 to -3.35) | -31.6% |
| Southern Sub-Saharan Africa | 345904 (313566 to 379466) | 469094 (426794 to 514923) | 779.84 (712.27 to 852.57) | -1.18% (-1.69 to -0.67) | 35.6% |
| Tropical Latin America | 1375724 (1321461 to 1449016) | 1185846 (1120648 to 1249061) | 479.13 (452.22 to 504.9) | -3.77% (-3.88 to -3.66) | -13.8% |
| Western Europe | 2234959 (2152606 to 2314268) | 1481012 (1356134 to 1575799) | 169.13 (158.16 to 178.61) | -3.34% (-3.48 to -3.21) | -33.7% |
| Western Sub-Saharan Africa | 1844345 (1534588 to 2202667) | 2963762 (2462104 to 3538782) | 1348.29 (1143.33 to 1588.36) | -1.09% (-1.17 to -1) | 60.7% |
